# Dual production of turquoise hydrogen and hybrid carbon nano materials from plastic waste over Co-Mo/Al-Mg catalysts

**DOI:** 10.1038/s41598-026-66503-3

**Published:** 2026-08-19

**Authors:** Ahmed M. Haggar, Ahmed E. Awadallah, Ateyya A. Aboul-Enein, Galal H. Sayed

**Affiliations:** 1 Process Development Division, Egyptian Petroleum Research Institute, Nasr City, 11787 Cairo Egypt; 2 Chemistry Department, Faculty of Science, Ain Shams University, Cairo, Egypt

**Keywords:** Plastic waste, Hydrogen, Carbon nanomaterials, Al_2_O_3_-MgO mixed oxides, Hybrid materials, Environmental sciences, Energy science and technology, Materials science, Nanoscience and technology

## Abstract

An optimized Co–Mo/Al₂O₃–MgO catalyst composition was developed to achieve stable and prolonged catalytic performance for the production of turquoise hydrogen from low-density polyethylene (LDPE) waste while simultaneously tailoring the morphology of the resulting carbon nanomaterials. To investigate the influence of support composition on catalytic performance, a series of Al₂O₃–MgO binary oxide supports with different weight ratios (Al75:Mg25, Al50:Mg50, and Al25:Mg75) were synthesized. The prepared supports and their corresponding fresh and spent catalysts were comprehensively characterized using FTIR, Raman spectroscopy, X-ray diffraction (XRD), BET surface area analysis, transmission electron microscopy (TEM), and thermogravimetric analysis (TGA). Among the Al₂O₃–MgO mixed-oxide-supported catalysts, Co–Mo/Al_25_–Mg_75_ exhibited the highest hydrogen concentration and produced well-graphitized multi-walled carbon nanotubes (MWCNTs). In contrast, Co–Mo/Al_75_–Mg_25_ exhibited the lowest hydrogen production and promoted the formation of turbostratic carbon, graphitic flakes, carbon nanofibers (CNFs), dense graphene layers, and carbon nano-onions (CNOs) encapsulating Co particles. Although CoMo/MgO exhibited the highest hydrogen concentration overall among all the investigated catalysts, Co–Mo/Al_25_–Mg_75_ provided the best overall catalytic performance when hydrogen production was considered together with the yield and graphitization quality of the carbon nanomaterials. These findings demonstrate that optimizing the Al₂O₃–MgO support composition effectively balances hydrogen production and carbon nanomaterial growth during catalytic conversion of plastic waste.

**Supplementary Information:**

The online version contains supplementary material available at 10.1038/s41598-026-66503-3.

## Introduction

Currently, the world’s population stands at 7.7 billion people, with a projected increase to 9 billion by 2050. If current human tendencies continue, it is estimated that the earth would be unable to accommodate more than 10 billion people^1^. The demand for plastic production is expected to reach 500 million tons by 2025 and to quadruple by 2050^2^. Plastic products are widely used in all walks of life due to their low production costs and convenient properties such as lightweight, low density, corrosion resistance, durability, and durability. Considered together, emissions of greenhouse gases, particularly CO_2_, were estimated to be 5.1 tCO_2_/t plastics^3^.

On a global scale, 6.9 billion tons of the approximately 9.1 billion tons of solid waste are plastic waste^4^. As a result, the buildup of plastic waste in the environment has negative effects on not just biological systems including the air, terrestrial habitats, marine life, and water supplies but also on the economy and causes issues with both human and animal health. Plastic garbage could spread diseases to people by sheltering insects like mosquitoes. Besides these issues, using plastics for food and beverage preservation can lead to cardiovascular system damage, cancer, adult-onset diabetes, early puberty, obesity, and resistance to chemotherapy^4^. Recent research focuses on the potentially dangerous impact of plastic trash buildup on COVID-19 infections^5^. Additionally, more than 79% of these plastic wastes were carelessly dumped in landfills or the environment, while just 9% were recycled and 12% were incinerated. In fact, 5 million tons of plastic trash were mechanically recycled and around 50,000 tons of plastic waste were chemically recycled^2^. Therefore, effective and affordable management techniques for plastic garbage are required.

African nations with vast populations, such as Egypt, Nigeria, and South Africa, generate more trash and emit more greenhouse gases. Particularly, plastic bags have a detrimental effect on the environment, agriculture, tourism, and the economy. For instance, it was stated that over 3000 plastic bags were filled within one kilometer of the most popular tourist destinations in South Africa^4^. According to Cudjoeb and Acquah^6^, Egypt may have 100,125.68 k tons of CO_2_eq to record the highest global warming emissions in Africa by 2025. In Egypt, plastic trash reached 13% of solid garbage, although there aren’t any recent, credible statistics available on waste generation and management. It’s interesting to note that Egypt, the largest consumer of polymers in Africa in 2017, used around 2.1 million tons of them^7^. In addition, the most common plastic types produced and used in Egypt include low-density polyethylene (LDPE), high-density polyethylene (HDPE), polypropylene (PP), and polyethylene terephthalate (PET). Furthermore, garbage creation is more dependent on metropolitan areas like Greater Cairo than it is on rural areas like the Delta and Upper Egypt. The predicted annual increase in garbage generation in Egypt from 2000 to the present is greater than 36%.

In order to recover the energy content and material value from these wastes, one viable option is to convert plastic trash into unique carbon nanomaterials (CNMs) and alternative green energy carriers (hydrogen) using green technology. Contrary, green hydrogen has been created photo-catalytically through water splitting in a number of investigations^8,9^. However, further research is needed to address the economic and productivity challenges of these green technologies.

The present approach relies on the thermal cracking of plastic waste to produce gaseous products that serve as a cheap and environmentally friendly source of both hydrogen and CNMs rather than their conventional supplies without any further separation. It’s interesting to note that these gaseous hydrocarbons further undergo catalytic decomposition over specific catalysts, particularly transition metals. Numerous researchers investigated different transition metals like Fe, Co, and Ni with varied promoter compositions such as Mo, Cr, Mn, Cu, and others over a variety of supports like Al_2_O_3_, MgO, SiO_2_, TiO_2_, and others^10–16^. Investigations into the thermal catalytic decomposition of methane (TCD) have focused on metal loading, preparation techniques, pretreatment conditions (calcination and reduction), and metal support interactions^17–19^. Presently, CoMo catalysts basically over MgO and Al_2_O_3_ have been evaluated for TCD. Great efforts were motivated to enhance their catalytic performance including metal content, preparation methods, and pretreatment process (calcination and reduction)^20^. Although Al_2_O_3_ possesses the right mechanical qualities, chemical stability, and large specific surface area^21^, it also releases inactive cobalt aluminate that is poorly reducible and has little interaction with Mo, which accumulates on the surface^21,22^. On the other hand, MgO has a strong propensity to absorb moisture and CO_2_ onto its surface and sublayers. During impregnation, MgO transforms into Mg(OH)_2_, which is then retained as MgO with various textural qualities following calcination^21^. Due to its basic nature and lower electronegativity than amphoteric Al_2_O_3_, MgO is, in this regard, more reactive toward metals. As a result, MgO facilitates the dispersion of loaded metals by interaction with the support’s metal. Moreover, the ease with which Co dissolves in MgO improves the creation of a solid solution. It was claimed that adding MgO changed the textural characteristics of Al_2_O_3_^23^. Additionally, choya et al.^23^ examined the beneficial effects of adding MgO in Co/Al_2_O_3_ for methane combustion because MgO coverage of Al_2_O_3_ support limits the formation of inactive phase CoAl_2_O_4_. For instance, the composition of the support has a significant impact on the entire catalyst’s catalytic activity. It is crucial to show the composition, distribution, and reducibility of the active species. Therefore, in order to produce a high catalytic effect, it is essential to develop catalysts with the appropriate support composition. In several catalytic processes, supports with both Al_2_O_3_ and MgO structural composition have been studied to see how well they affect the catalyst activity^21–35^. In our reaction, both bare MgO and Al_2_O_3_ have been studied for CoMo catalysts^12,13,36–40^ and they showed better efficiency in TCD. According to our knowledge, few studies are available on the influence of Al_2_O_3_-MgO mixed oxides on the CoMo catalyst in TCD. Theoretically, support with synergist structural function may result in promising catalytic action as it combines dual merits of Al_2_O_3_ and MgO, or reduce the limitation of each bare support. For the co-production of hydrogen and CNMs, successive trends toward plastic wastes, particularly LDPE and PP, are now being applied^17,41,42^. In our earlier research^42^, we assessed how the total metal content of Co-Mo over MgO support affected the catalytic activity. On the basis of this investigation, we would add a different weight ratio of a different support, such as Al_2_O_3_, to the MgO support. Additionally, we would look at how well the Co-Mo catalysts worked with Al_2_O_3_-MgO mixed oxides in terms of their performance with Al_2_O_3_ as well as their performance with MgO in our earlier research^42^.

Here, a variety of supports made of bare Al_2_O_3_, bare MgO, and their mixed oxides with various content would be created and examined as supports for a Co-Mo/support catalyst with a composition of 4:1:5. The impact of bare support and their binary oxides on catalytic behavior with regard to both hydrogen generation and CNMs growth would be assessed. Depending on the support composition, CNMs with different morphologies and structures have been produced, including tubular CNTs, ribbon CNFs (r-CNFs), bamboo CNTs (b-CNTs), fishbone CNFs (f-CNFs) or herringbone CNFs, graphitic, carbon nano-onions (CNOs), platelet-like CNFs (P-CNFs), and turbostratic CNFs (t-CNFs) with different composites. Furthermore, based on the support composition, new growth paths for the developed CNMs have been proposed. Subsequently, a correlation has been shown between the produced CNMs for each support composition and the hydrogen productivity.

## Experimental

### Catalyst preparation

Using the prepared supports (Supplementary information), Co: Mo: Support catalysts with a weight ratio of 4:1:5 were produced over Al_2_O_3_, Al_75_-Mg_25_, Al_50_-Mg_50_, Al_25_-Mg_75_, and MgO. Each designed support was also treated with the calculated amounts of cobalt nitrate [Co(NO_3_)_3_.6H_2_O] and ammonium heptamolybdate [(NH_4_)_6_Mo_7_O_24_] adequate aqueous solutions (Sigma-Aldrich), which were then sonicated for 25 min while being combined on a stirrer at 100 °C. Pasty components were completely dehydrated, desiccated at 120 °C, and then calcined for 4 h at 600 °C.

### Characterization techniques

Fresh catalysts and CNMs products were both examined using powder X-ray diffraction (XRD) measurements on an X’Pert PROPANalytical equipment with CuKa radiation (k = 0.1541 nm) and a 2 range from 4° to 90°. Crystallite sizes are measured using the Debye-Scherrer equation, which is given as (D = k/cos), where D, λ, β, θ and (FWHM) are the crystallite size, wavelength, diffraction angle, and full width at half maximum, respectively. Moreover, all of the produced catalysts and the as-grown CNMs were examined using a SENTERRA Dispersive Raman Microscope (Bruker) equipped with a diode Nd: YAG laser with a 532 nm wavelength and a Raman shift (100-3,000 cm^-1^). Fourier transform infrared (FTIR) analysis was carried out on fresh catalysts using a PerkinElmer spectrometer model 100 series to reveal the functional groups on the catalyst surface.

The reducibility and metal-support interaction were investigated using BELCAT-II chemisorption apparatus (BEL Inc., Japan). Within 10 °C/min of a continuous heating rate, temperature programmed reduction (TPR) from 50 °C to 1,000 °C was carried out in a quartz reactor using 5%H_2_/Ar_2_ flow (30 ml/min). In this regard, N_2_ adsorption/desorption isotherms of fresh catalysts were recorded at liquid nitrogen temperature using a programmed physical-sorption equipment (Autosorb-1 C, Quantachrome Instruments). Initially, the catalysts were degassed at 200 °C for 3 h in a vacuum. Both the specific surface area and the pore size distribution were determined using the BET method and the Barrett-Joyner-Halenda (BJH) method.

The type, macro, and microstructures of the as-grown CNMs were displayed using a transmission electron microscope (TEM, Model JEM-200CX, JEOL, Japan). Each CNM sample was dissolved in a reasonable amount of ethanol and sonicated for 20 min. Droplets of suspended material were positioned on a carbon cover grid and then put inside a TEM to be imaged. Furthermore, both differential thermogravimetric analysis (DTG) and thermogravimetric analysis (TGA) were performed using the SDT; Q600 apparatus with the necessary amount of each sample and a heating ramp of 10 ^ᶱ^C/min in an airflow of 50 cm^3^/min.

## Results and discussion

### Characterization of fresh supports and their fresh catalysts

#### FTIR

**Fig. 1:**
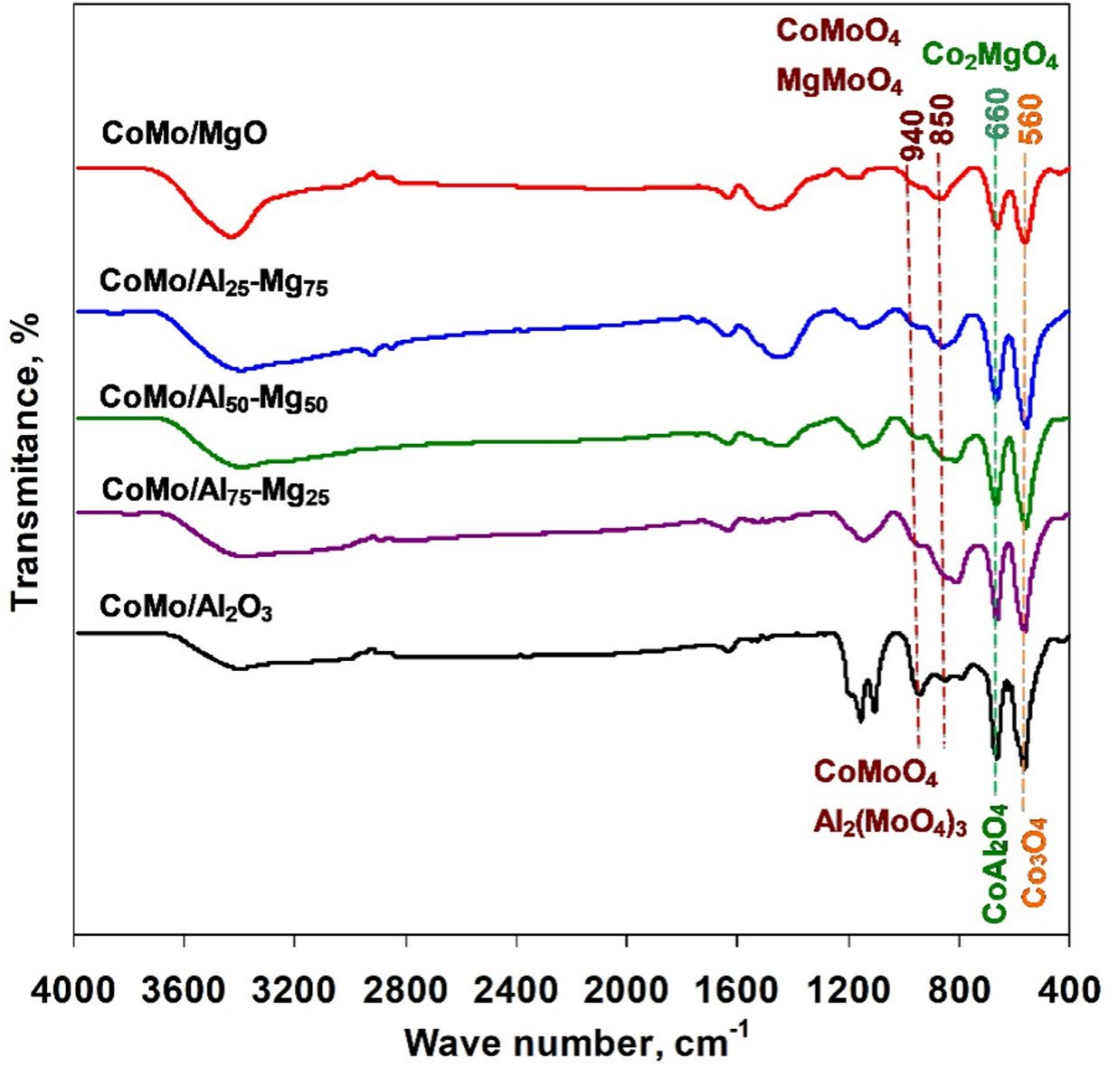
FTIR spectra of fresh calcined Co-Mo catalysts.

To examine the surface structure and bonding nature after depositing of bimetallic (Co and Mo) on the investigated supports, FTIR spectra are represented in Fig. 1. The recognizable bands of alumina and magnesia in shrink or vanish when the Co-Mo bi-metals are added to Al_2_O_3_ and MgO (Fig. 1). Additionally, for all catalysts, new bands below 1000 cm^-1^ are identified at about 560, 660, 850, and 940 cm^-1^. In detail, 560 and 660 cm^-1^ bands are ascribed to octahedral and tetrahedral Co-O transmissions in Co_3_O_4_, which implies the presence of non-reacted Co_3_O_4_ on the surface^43^. The well-resolved band at 660 Cm^-1^ can also be attributed to the tetrahedral Co-O in CoAl_2_O_4_^44^ and the MgO_4_ tetrahedron^45^, which raises the possibility of CoAl_2_O_4_ and Co_2_MgO_4_ formation in CoMo/Al_2_O_3_-MgO catalysts. Furthermore, bands at approximately 850 and 940 cm^-1^ can show how molybdenum species including CoMoO_4_, MgMoO_4_, and Al_2_(MoO_4_)_3_ form in CoMo/Al_2_O_3_-MgO catalysts. It is clear that the characteristic band of the molybdenum mixed metal oxide species, 850 cm^-1^, is weakened by alumina, not by magnesia. Additionally, adding magnesia to an alumina support enhances the molybdenum species band. This pattern shows that MoO_3_ prefers to interact with MgO over Al_2_O_3_.

#### Raman spectroscopy

**Fig. 2:**
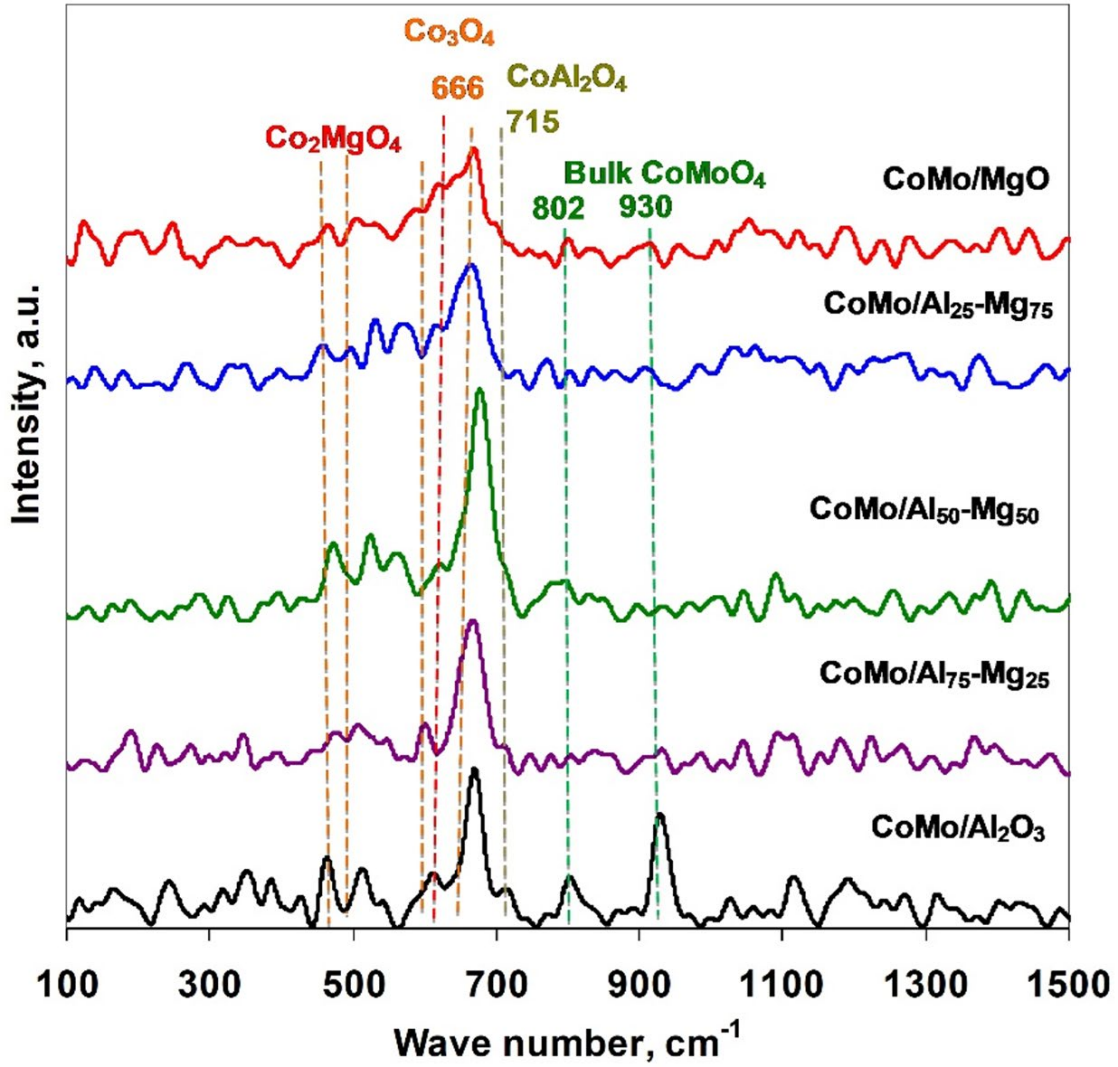
Raman spectra of fresh calcined CoMo catalysts.

Despite the fact that alumina and magnesia are inactive for Raman spectroscopy^22^, all of the prepared catalysts are examined by Raman (Fig. 2). Indeed, Raman is employed to determine how the support composition affects the catalyst surface and how various species, including Co_3_O_4_, MoO_3_, Al_2_O_3_, and MgO, interact with one another (Fig. 2). Firstly, the absence of MgO signal can be attributed to fluorescence problematic where only α-Al_2_O_3_ type could be Raman characterized^26^. All of the prepared catalysts exhibit a clean signal at approximately 666 cm^-1^. This signal may be associated with Co_3_O_4_^23^ and may show the accumulation of non-interacted Co_3_O_4_ on whole supports with different combinations. Further signals are found for the CoMo/Al_2_O_3_ catalyst at about 802 and 930 cm^-1^ as well as the well-shoulder at 715 cm^-1^ (Fig. 2). Thus, the appearance of signals at 802 and 930 cm^-1^ could serve as an illustration of how bulk CoMoO_4_ forms on surfaces^46^. Furthermore, the shoulder at 715 cm^-1^ would support the existence of CoAl_2_O_4_^23^. This shoulder shifts and shrinks when magnesia is assimilated to alumina. It could be made clearer that adding MgO to the structure causes less CoAl_2_O_4_ to form. Additionally, as the MgO content increases, the distinctive signals of bulk CoMoO_4_ species vanish. On the other hand, CoMo/MgO and CoMo/Al_25_-Mg_75_ catalysts exhibit a very broad signal that is shifted to a lower wavenumber at 660 cm^-1^. Meanwhile, it is anticipated that increasing the magnesia concentration may favor the interaction of Co_3_O_4_ and MgO. It is evident that as the alumina content of CoMo/Al_2_O_3_-MgO mixed oxides increases, the signal at 660 cm^-1^ sharpens and shifts to a higher wave number. Signals at 475, 510, and 611 cm^-1^ are also discernible. It becomes clear that rising alumina content encourages the surface’s capacity to assemble free Co_3_O_4_. Based on the aforementioned, MgO has a preference over alumina toward Co_3_O_4_ and MoO_3_ to form interacted species of their mixed oxides. Alumina would therefore reduce the distribution of impregnated species such as Co_3_O_4_ and MoO_3_ on the supports with the binary oxides structure.

#### XRD diffractions

**Fig. 3:**
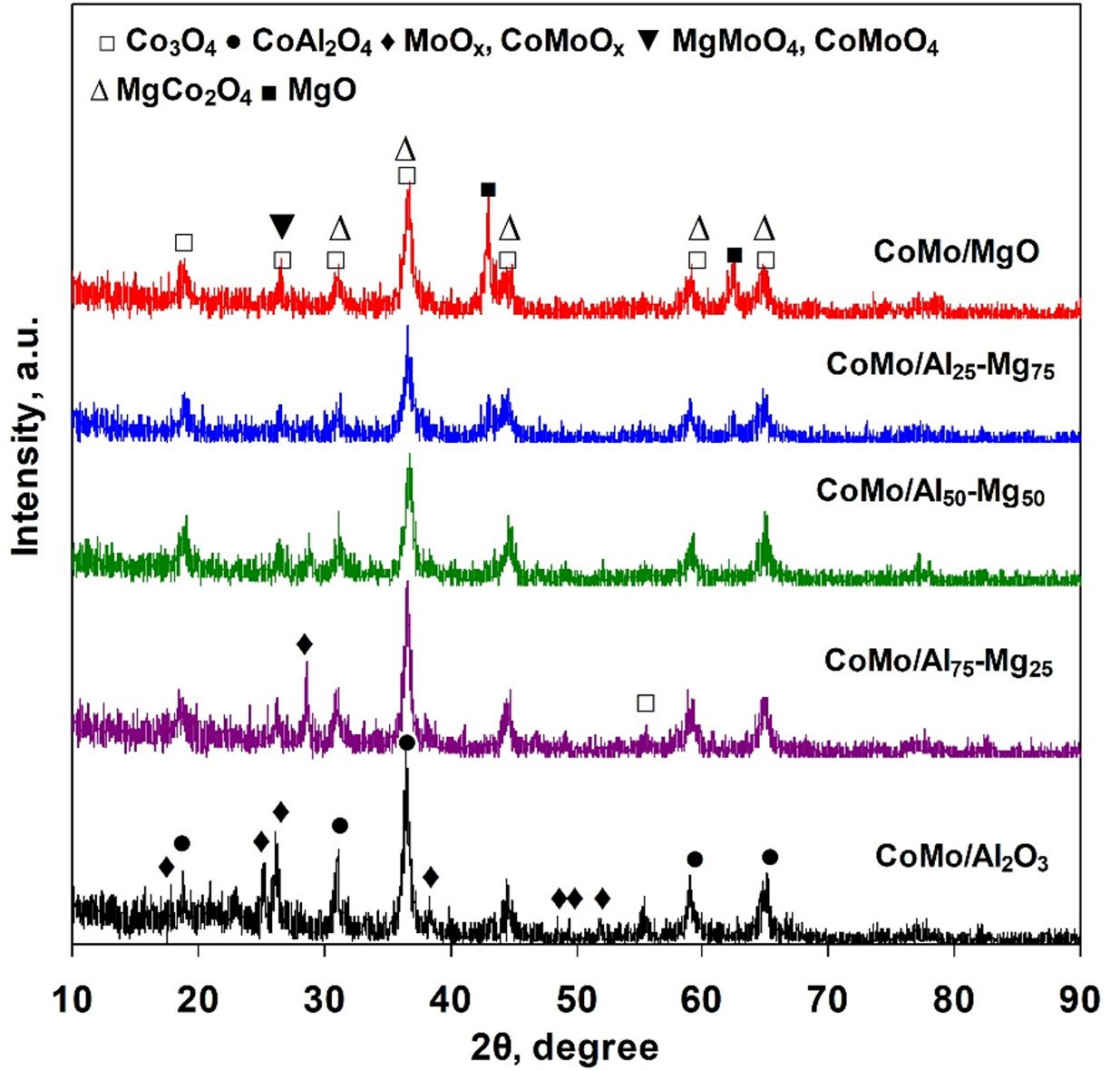
XRD patterns of fresh calcined CoMo catalysts.

Figure 3 depicts the crystallographic transformation of the freshly calcined catalysts after loading the active bimetallic components, cobalt oxide and molybdenum oxide, on the examined supports. All of the fresh catalysts exhibit comparable diffraction phases with varying intensities and broadness at 19°, 31°, 36°, 44.5°, 55.2°, 59°, and 65°, which can be attributed to Co_3_O_4_ on the surface of all the investigated supports^30^. In the case of CoMo/Al_2_O_3_, Co_3_O_4_ and CoAl_2_O_4_ have similar overlapped phases, making it very challenging to distinguish between them by XRD^18,25^. However, Liu et al.^31^ noted that both phases at 31° and 59° are distinctive peaks for CoAl_2_O_4_ on the Al_2_O_3_ surface. It is also evident that molybdenum oxides and their composites are present on the surfaces of CoMo/Al_2_O_3_ and CoMo/Al_75_-Mg_25_ within 2Ө = 23°, 25°, 26°, 28.6°, 38°, 48.5°, 49.5°, and 53.3°^47–50^. Furthermore, it is possible to hypothesize that the insufficient calcination temperature would prevent the evolution of Al_2_(MoO_4_)_3_. Furthermore, Shaheen and Abd El Maksod^20^ reported that it forms between 750 and 1,000 °C during calcination. Furthermore, the finding that the formation of aluminum molybdate was prevented by the presence of cobalt oxide^51^ provided further support. For CoMo/MgO and CoMo/Al_25_-Mg_75_ catalysts, a signal at about 26.6° is noticed after adding MgO to the catalyst successively. This signal may come from molybdenum composites such as MgMoO_4_ and CoMoO_4_^52^. In addition, MgO peaks are also observed at about 43° and 62.5° for the CoMo/MgO and CoMo/Al_25_-Mg_75_ catalysts^23^. Furthermore, it has been found that MgO weakens the Co_3_O_4_ signals. XRD results show that magnesia increases the distribution of both Co_3_O_4_ and MoO_3_ due to its propensity to form several binary mixed metal oxides, including MgMoO_4_, Co_2_MgO_4_, and CoMoO_4_. On the other hand, alumina has a low affinity for both Co_3_O_4_ and MoO_3_, which increases the chance of bulk CoMoO_4_ formation as well as the accumulation of free Co_3_O_4_ and MoO_3_ on the surface.

#### H_2_-TPR measurements

**Fig. 4:**
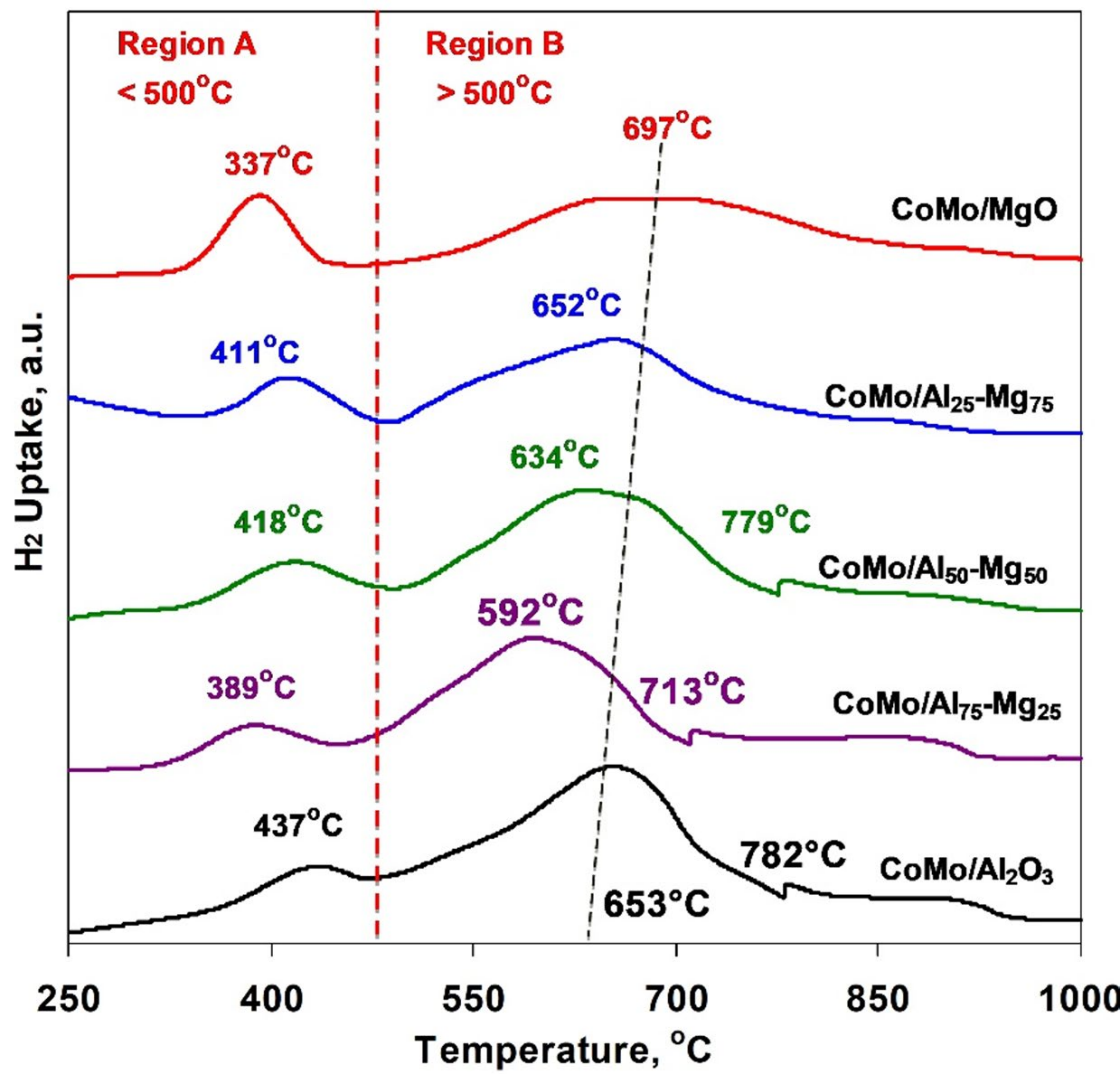
TPR profiles for fresh calcined CoMo catalysts.

Figure 4 displays the hydrogen temperature-programmed reduction profiles of CoMo-based catalysts. All the catalysts show mainly two reduction areas at different reduction temperatures. The first reduction area at lower temperatures would be for surface Co_3_O_4_^23^, whereas the second reduction area at higher temperatures would be for interacting Co_3_O_4_ species with other neighbors^22^. Depending on the type of interacting species, degree of interactions, and support composition, the reduction regime would be defined. To investigate the reduction behavior of each catalyst with mixed alumina-magnesia composites, both bare alumina and bare magnesia-supported catalysts’ reduction trends should be elucidated. Firstly, CoMo/Al_2_O_3_ and CoMo/MgO catalysts exhibit peaks before 500 °C at 437 °C and 391 °C, respectively, which attribute to accumulated non-interacting Co_3_O_4_^53^. Thus, MgO facilitates the reduction of surface Co_3_O_4_ particles rather than Al_2_O_3_. The second reduction region for the CoMo/Al_2_O_3_ catalyst is also moved to a lower temperature range at 653 °C, and another shoulder is noticed at a higher temperature range of 782 °C (Fig. 4). This second reduction area confirms the formation of inter-acting species such as large particles of Co_3_O_4_^25^, CoMoO_4_^48^, Co_2_AlO_4_, and CoAl_2_O_4_^18,22^. The reduction behavior of Co_3_O_4_ is claimed to be simpler than that of Co_2_AlO_4_ or CoAl_2_O_4_^18^.

Contrarily, the CoMo/MgO catalyst is directed to a higher wide temperature regime at 697 °C (Fig. 4). Also, this higher temperature area refers to the formation of inter-acting species with different strengths, such as MgMoO_4_^21^, CoMoO_4_, and Co_2_MgO_4_. Özdemir and Öksüzömer^35^ stated that hard reducibility areas indicated species with strong metal-support interactions. Due to the formation of mixed oxide species like MgMoO_4_ and MgCo_2_O_4_, MgO hardens the second stage of reduction and improves the distribution of bi-metals (Co, Mo)^49^. This suggests that Co_3_O_4_ interacts with MgO more strongly than Al_2_O_3_^23^. It is obvious that MoO_3_ reacts strongly with MgO to produce MgMoO_4_ rather than Al_2_O_3_ to produce Al_2_(MoO_3_)_4_^21^. Moreover, it was claimed that MgMoO_4_ required a higher temperature to be reduced than MoO_3_^21^. Also, the reduction of molybdenum species may contribute to the catalyst’s reducibility in stages, i.e., octahedral Mo^6+^→octahedral Mo^4+^ and tetrahedral Mo^6+^→tetrahedral Mo^4+^ in the first reduction area, where octahedral and tetrahedral Mo^4+^→Mo^0^ in the second reduction area^24,33^. In the same way that MoO_3_ improves Co_3_O_4_ dispersion, Co_3_O_4_ also makes MoO_3_ more reducible^48^. This is in line with XRD, which supports MgO’s high affinity for Co_3_O_4_ and MoO_3_, as well as Al_2_O_3_’s weak affinity for MoO_3_ and good affinity for Co_3_O_4_.

The CoMo/Al_75_-Mg_25_ and CoMo/Al_25_-Mg_75_ catalysts are reduced at lower temperatures than their pure supported catalysts (CoMo/Al_2_O_3_ and CoMo/MgO), demonstrating how each dopant contributes structurally to the bulk support (Fig. 4). In the case of the CoMo/Al_75_-Mg_25_ catalyst, MgO hinders Co_3_O_4_ and Al_2_O_3_ interactions, diminishes the formation of Co-Al species, and incentivizes the agglomeration of extra-large Co_3_O_4_ particles and the avidity to form more CoMoO_4_. Al_2_O_3_ inclusion in MgO is also seen to shift the reduction temperature to a lower one. It is obvious that the alumina dopant serves as a barrier and lessens interaction between Co_3_O_4_, MoO_3_, and MgO. Notably, adding increasing amounts of MgO to the matrix allows for the generation of Co, Mo, and MgO-interacting species in addition to inhibiting Co-Al species. This suggests that the CoMo/Al_25_-Mg_75_ is less reducible than the CoMo/Al_75_-Mg_25_. These findings support the Raman data and are consistent with earlier literature^23^. It is believed that it is impossible to prove the existence of MgO-based solid solutions (Co, Mg) O and Mg(Co, Al)O in CoMo/Al_2_O_3_-MgO catalysts because they are reduced at higher temperatures^54^. Furthermore, Table 1 summarizes all the reduction information for all the examined fresh catalysts, including hydrogen consumption, reduction areas, and reduction temperatures.

**Table 1:** Hydrogen consumption data during reduction behavior of fresh calcined CoMo catalysts supported over bare and binary supports.

Catalyst	H_2_ Consumption, m mole/g		Total H_2_ Consumption (m mole/g)
	˂500 °C	˃500 °C	
CoMo/Al_2_O_3_	2.6	12.4	15
CoMo/Al_75_–Mg_25_	1.6	9.9	11.5
CoMo/Al_50_–Mg_50_	3.3	10.7	13.9
CoMo/Al_25_–Mg_75_	1.4	5.9	7.3
CoMo/MgO	2.3	8.8	11.1

#### Texture characteristics

**Fig. 5:**
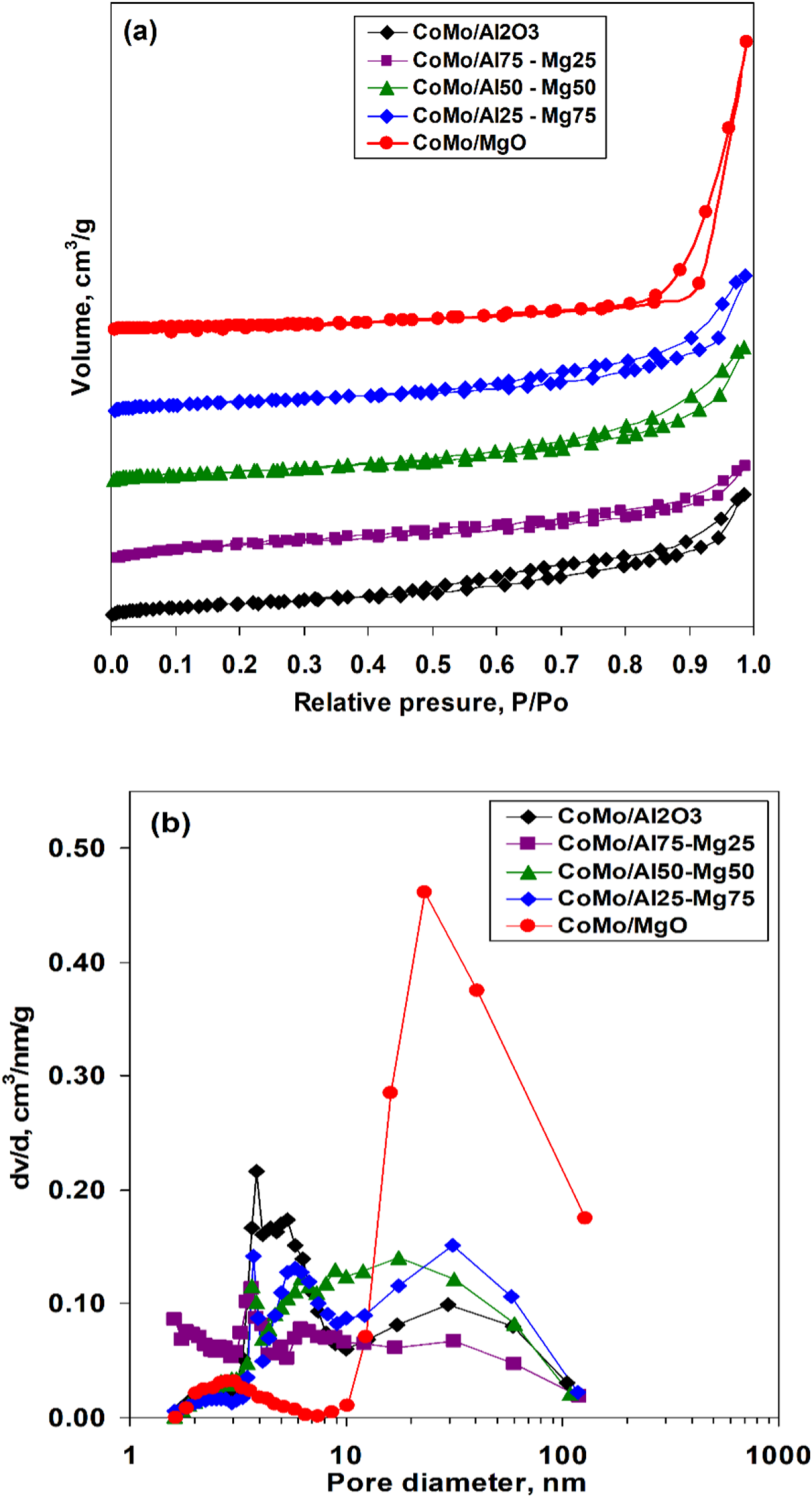
**(a)** Nitrogen adsorption-desorption isotherms and **(b)** pore size distribution curves for Co-Mo catalysts.

Figure 5 displays the nitrogen adsorption-desorption isotherms and pore size distribution curves for every prepared catalyst. According to IUPAC classification, all prepared catalysts exhibit isotherm type IV with the exception of CoMo/Al_75_-Mg_25_ catalyst, which resembles isotherm type II without a hysteresis loop or an incredibly narrow loop. Interestingly, isotherm type IV resembles isotherm type II with a hysteresis loop. Cychosz and Thommes^55^ reported that materials with pores wider than 4 nm would have hysteresis loops. All the isotherms have monolayer and multilayer regions without plateau regions, excluding the CoMo/MgO catalyst; it shows the previous three regions with hysteresis loop H3. All catalysts with isotherms of type IV confirm the meso-porosity existence and capillary condensation adsorptions, while type II with a narrow loop relates to mainly non–porous materials with poorer meso-porosity^40^.

Configuration of the hysteresis loop H3 indicates the mesoporous’ morphology to be slit-shaped pores and happens with aggregates of slit-like particles^56,57^. Additionally, width, length, closure point, steepness, and direction of desorption to adsorption reveal more information about pores. It is clarified that the hysteresis loop closes at a relative pressure of p/*p* = 0.4–0.6 for all catalysts except CoMo/MgO, and its closure is noticed at p/*p* = 0.8 with steeper action, i.e., adsorption and desorption are vertical and parallel (Fig. 5). Hence, shifting to higher relative pressures signifies larger pore diameters^58^. Unexpectedly, it was stated that hysteresis loop H3 could identify macro pores in networks even when pore condensate was only partially filled^59^. Additionally, the closure point at lower relative pressures is associated with mesopores with smaller diameters^58^. As well as, it was stated that a wider hysteresis loop revealed a longer pore length. Hysteresis loop width is used to gauge the degree of meso-porosity in a structure, so the CoMo/Al_75_-Mg_25_ catalyst would have the fewest mesopores due to the detrimental effects of the incorporated MgO. While adding more MgO causes curvatures to widen and mesoporous structures to increase (Supplementary Fig. 5a) efforts^39^.

As a result, the adsorption-desorption behaviors are controlled by the geometries of the pores, including shape, diameter, volume, individual, and networks. The effect of support composition on pore size distribution is investigated (Fig. 5b). Notably, all catalysts have nanoscale pores, or pores that are less than 100 nm^59^. Both CoMo/Al_2_O_3_ and CoMo/MgO catalysts have been observed to have bimodal pore distributions, which are composed of small and large mesopores. Additionally, the CoMo/Al_2_O_3_ catalyst exhibits narrower mesopores than the larger one. Contrarily, it appears that CoMo/MgO has more macro pores and larger mesopores with a wider distribution than narrow mesopores. According to percolation action, CoMo/Al_75_-Mg_25_ exhibits so few narrower mesopores that resemble the mono-modal distribution range. With an increase in MgO content, both wide and narrow mesopores develop. The aforementioned textural findings corroborate all the complementary information from XRD, Raman, and FTIR.

### Catalytic activity and durability

**Fig. 6:**
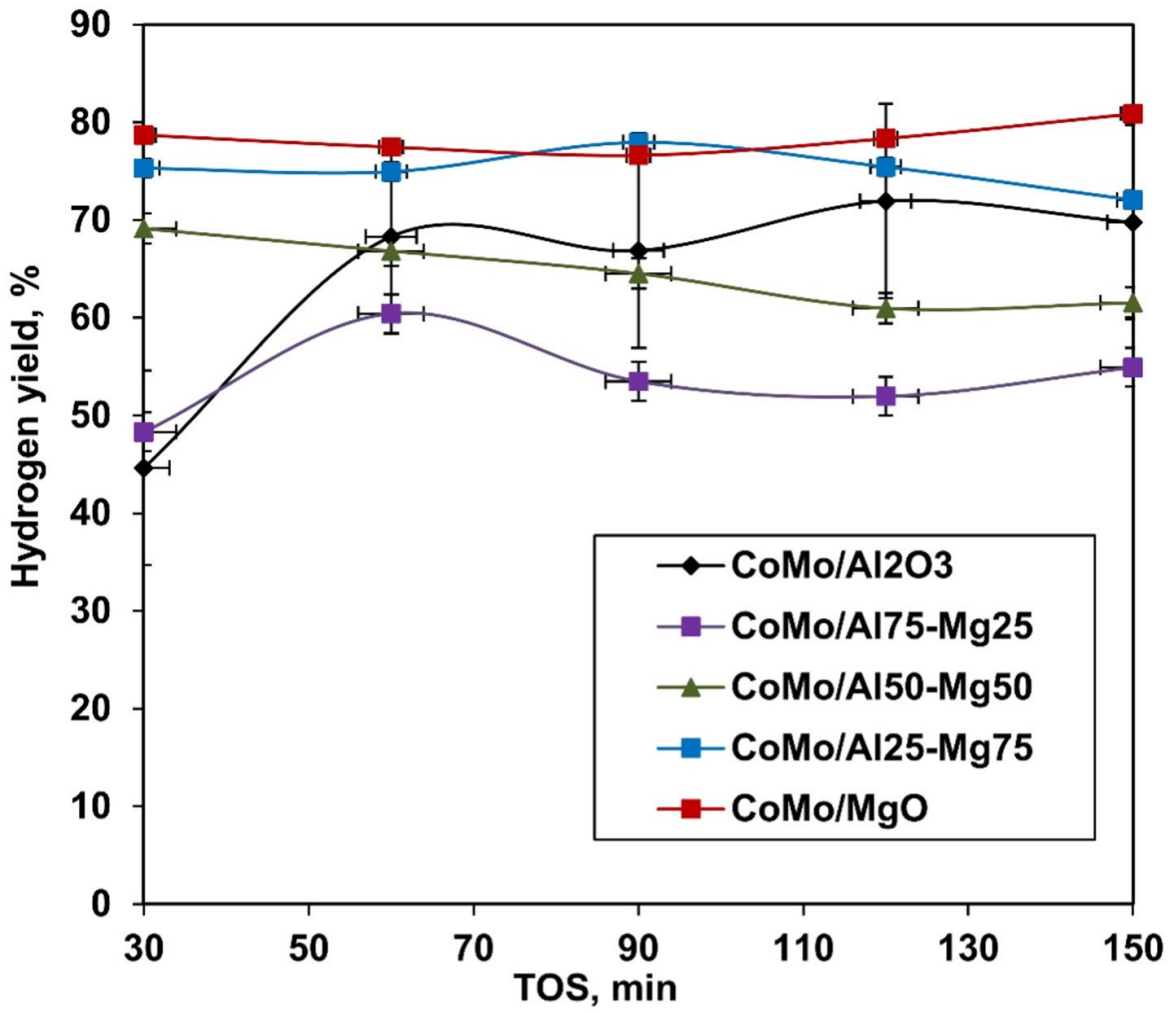
Hydrogen concentration as a function of time on stream (TOS) . for Co-Mo catalysts.

Figure 6 demonstrates the catalytic performance of the prepared catalysts with different support combinations by plotting hydrogen concentration as a function of time on stream (TOS) of 150 min. All the catalysts show different catalytic activity toward conversion of the same inlet hydrocarbons to different hydrogen concentrations with variable purities and notable deviation in their catalytic behaviors, such as their initial-final hydrogen concentration, deactivation, stability, and prolonged activity during the reaction progress. Regarding hydrogen production, CoMo/MgO exhibited the highest hydrogen concentration throughout the entire reaction period, maintaining stable catalytic activity without noticeable deactivation. CoMo/Al_25_–Mg_75_ showed comparable hydrogen-production performance, although a slight decline in hydrogen concentration was observed after 90 min of reaction. CoMo/Al_2_O_3_ produced lower hydrogen concentrations than CoMo/MgO and CoMo/Al_25_–Mg_75_ but outperformed CoMo/Al_75_–Mg_25_ and CoMo/Al_50_–Mg_50_ over most of the reaction period. Notably, CoMo/Al50–Mg50 initially generated a higher hydrogen concentration than CoMo/Al_2_O_3_ during the first 30 min, reaching 69 vol%, before gradually declining as the reaction progressed. These findings identify CoMo/MgO as the most effective catalyst for sustained hydrogen production under the investigated conditions. However, the overall catalytic performance was evaluated based on the combined hydrogen-production and carbon nanomaterial-production results, as discussed in the following sections.

In contrast, CoMo/Al_2_O_3_ has a low initial hydrogen concentration but performs better catalytically than both CoMo/Al_75_-Mg_25_ and CoMo/Al_50_-Mg_50_ over the course of the reaction. It clearly shows that CoMo/Al_75_-Mg_25_ had the lowest hydrogen concentration during TOS (Fig. 6). Regarding Fig. 6, the hydrogen output, or the ability to convert the hydrocarbons at the inlet into hydrogen, could be used to determine the catalytic activity of these catalysts. As a result, the support’s composition would influence the hydrogen output and would regulate the catalytic behavior. MgO has a favorable effect on the cobalt and molybdenum active phases of the catalysts because of its strong affinity to interact and distribute with these molecules and create new phases from their mixed metal oxides. MgO plays a significant role in preventing Co_3_O_4_ agglomeration, preventing the active phases from sintering, controlling the size of cobalt nanoparticles, increasing the number of active sites that are accessible, promoting the breakdown of the inlet hydrocarbons, and improving hydrogen production. Even though mixed species of Co, Mo, and Mg are subjected to more difficult reduction conditions than free Co_3_O_4_, they regulate the catalytic performance by enhancing cobalt nanoparticle dispersion. In addition, Al_2_O_3_ interacts with cobalt oxides to create mixed oxides rather than MoO_3_. In this regard, fresh catalyst analyses confirm the formation of CoAl_2_O_4_, CoMoO_4,_ and large particles of Co_3_O_4_. Acomb et al.^41^ found that cobalt forms aluminates more readily than other metals like iron and nickel, but that the presence of molybdenum diminishes the crystallinity of both aluminates and Co_3_O_4_^60^. It is an indication of molybdenum’s role as an inhibitor in the formation of aluminates and extra-aggregation of Co_3_O_4_ that could occur at 600 °C^60^.

These findings confirm that MgO interacts more strongly with Co_3_O_4_ and MoO_3_ than Al_2_O_3_, which enhances the distribution of active phases and lessens sintering action. Wu et al.^61^ argued with the preferential role of MgO, which is attributed to its basic character and the low electronegativity of Mg^+ 2^ in MgO in comparison to Al^+ 3^ in Al_2_O_3_. Because of its basicity, MgO interacts more favorably with acidic MoO_3_, which can result in the formation of MgMoO_4_ not only during calcination but also during impregnation and drying of the prepared catalyst^21^. Where low electronegativity would abate the Mg-O bong and assist in being coordinated with loaded species like Co_3_O_4_. Rationally, for both Co_3_O_4_ and MoO_3_, it could arrange their reactivity toward their supports in the following order: MgO˃MgO-Al_2_O_3_˃Al_2_O_3_^62^. Therefore, the electronegativity of the support directs the catalytic activity toward hydrogen output. Taken into consideration, MoO_3_ evaporation may occur at about 790 °C, and amphoteric Al_2_O_3_ may prevent the formation of (Al, Mo) O interacted species at temperatures above 750 °C^20^. Despite having a larger S_BET_ (Supplementary Table S1) and more durable mechanical properties, Al_2_O_3_ produces less catalytic active phase than MgO. Thus, the catalytic decomposition of inlet hydrocarbons to produce hydrogen is a metal-catalyzed reaction, and textural characteristics like surface area are not the determining factor^63^. Moreover, the nature, quality, and number of accessible cobalt nanoparticle active sites dominate the reaction process.

The incorporation of Al₂O₃ into the MgO support modified the nature of the active phases and consequently influenced hydrogen-production performance. For the CoMo/Al_25_–Mg_75_ catalyst, the introduction of a limited amount of Al₂O₃ appears to provide multiple beneficial effects by improving the structural dispersion of Co₃O₄ and promoting the formation of additional CoMoO₄ species^20^. At the same time, it also promotes the formation of a limited amount of the less active CoAl₂O₄ phase, which may partially offset these beneficial effects. The balance between these competing processes likely accounts for the slightly lower hydrogen-production performance of CoMo/Al_25_–Mg_75_ compared with CoMo/MgO, while maintaining the highest performance among the Al₂O₃–MgO mixed-oxide-supported catalysts. In contrast, increasing the Al₂O₃ content further, as in CoMo/Al_75_–Mg_25_, is likely to weaken the beneficial interaction between MgO and the active phases, thereby promoting cobalt agglomeration through weaker metal–support interactions. Furthermore, the formation of MgMoO₄ may modify the distribution of Mo species by transferring a fraction of the Mo species from the catalyst surface into the bulk phase, thereby reducing the number of accessible active sites available for hydrocarbon decomposition. These combined effects are consistent with the lower hydrogen-production performance observed for CoMo/Al_75_–Mg_25_. The CoMo/Al_50_–Mg_50_ catalyst exhibited intermediate hydrogen-production performance (Fig. 6), indicating that the catalytic behavior of the Al₂O₃–MgO mixed-oxide-supported catalysts is governed by the balance between the favorable dispersion effect of MgO and the formation of less active mixed-oxide phases involving Al₂O₃. Overall, the results demonstrate that the support composition plays a critical role in regulating the interaction between the active phases and the support, thereby controlling hydrogen production and catalyst performance. However, it has been reported that not only is 600 °C a sufficient temperature for MgO and Al_2_O_3_ interaction as they are mixed with each other, but also MoO_3_ catalyzes their interactions^52^. This is despite supports and fresh catalyst analyses not fully confirming the formation of MgAl_2_O_4_. MgAl_2_O_4_ might make Co_3_O_4_ extra-segregation more likely by reducing the interaction between the metal and the support. According to the previously mentioned prediction, the development of larger cobalt nanoparticles was associated with poor interaction between Co_3_O_4_, MoO_3_, and Al_2_O_3_, which results in poor catalytic activity. Additionally, incorporating Al₂O₃ into the MgO support modifies the interaction between the Co and Mo active species and the support, which may partially diminish the favorable dispersion effect provided by MgO. Consequently, some Al₂O₃–MgO mixed-oxide catalysts exhibited increased cobalt agglomeration and a lower density of accessible active sites than CoMo/MgO, resulting in reduced hydrogen-production performance. In contrast, the CoMo/MgO catalyst exhibited stronger interactions among Co₃O₄, MoO₃, and MgO, leading to improved cobalt dispersion and the highest hydrogen-production performance under the investigated conditions. The well-dispersed cobalt nanoparticles are therefore considered to provide a greater density of accessible active sites for hydrocarbon decomposition and hydrogen generation. Incorporating Al₂O₃ into the MgO-supported catalyst may further improve the structural distribution of cobalt species; however, it may also promote the formation of less active cobalt aluminate phases, thereby partially offsetting the beneficial effect of improved dispersion. Consequently, the catalytic performance of the Al₂O₃–MgO mixed-oxide-supported catalysts reflects the balance between the enhanced dispersion provided by MgO and the formation of less active mixed-oxide phases involving Al₂O₃. This interpretation is consistent with the intermediate hydrogen-production performance observed for the mixed-support catalysts relative to CoMo/MgO.

### Characterization of the deposited CNMs

#### Raman spectroscopy

**Fig. 7:**
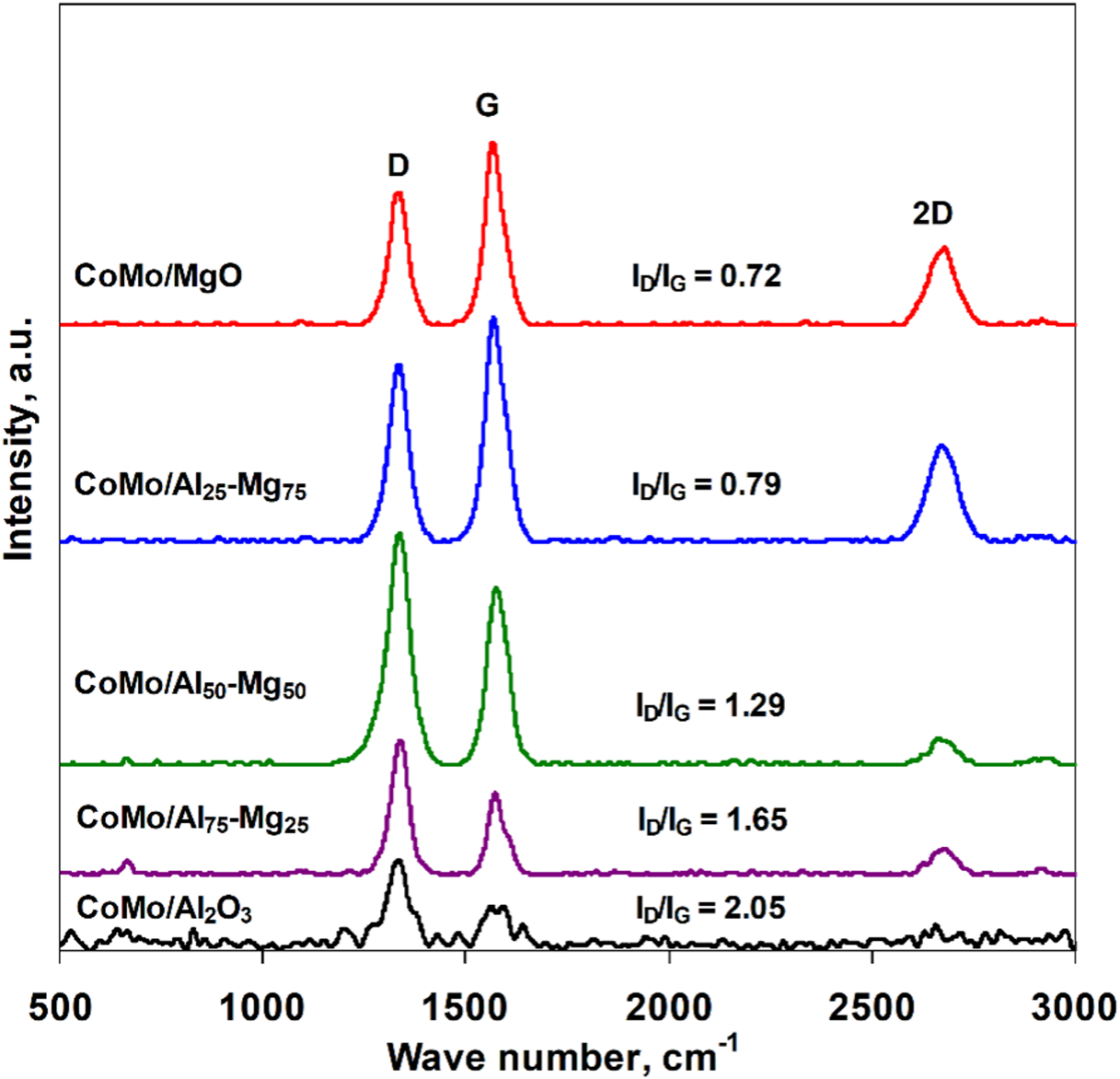
Raman spectra of as-deposit CNMs over CoMo catalysts.

Raman spectroscopy is a valuable nondestructive tool to differentiate well-ordered crystalline structures of CNMs from disordered structures. Figure 7 reveals the Raman spectra of all the used catalysts to characterize their quality and degree of graphitization. All spent catalysts rationally lost all of their catalyst bands before 1000 cm^-1^, implying the development of CNMs on their surfaces. For CoMo/MgO and CoMo/Al_25_-Mg_75_ catalysts, Fig. 7 clearly depicts highly ordered graphitization graphene walls that are related to the graphitization band at about 1580 cm^-1^ (G-band). In contrast, CoMo/Al_2_O_3_ and CoMo/Al_75-_Mg_25_ show more imperfections and distortions in their graphitic composition. The D band at 1350 cm^-1^ is responsible for these disorders, flaws, and distortions^64^. Notably, the presence of a 2D band at around 2750 Cm^-1^ is consolidated with increasing MgO content to reach its almost maximal intensity at CoMo/Al_25_-Mg_75_ and CoMo/MgO catalysts. The intense 2D band which involves two D band phonons with opposite, non-zero momenta^65^, is attributed to the highly oriented graphene wall deposition with better crystalline structure efforts^39^. It is also clear that the ID/IG ratios of their binary oxide-supported catalysts decrease with increasing MgO content (Fig. 7). It was reported that ID/IG ratios assess the quality and crystallinity of the as-grown CNMs^13^. The above-mentioned observations and variations in these Raman spectra are all supported by the TEM images in each case. In detail, turbostratic materials characterization in Figs. S.3 A-D, S.4 A-D, and S.5 confirm the higher I_D_/I_G_ values for CoMo/Al_2_O_3,_ CoMo/Al_75_-Mg_25_, and CoMo/Al_50_-Mg_50_ catalysts. Randomly overlapped dense graphitic layers with folded, wrinkled configurations (Figs. S.3–5) supports the poor quality of the Raman concept^66^. In Figs. S.3 and S.4, regularly ordered graphene walls guarantee better graphitization with the lowest ID/IG of the CoMo/MgO and CoMo/Al_25_-Mg_75_ catalysts.

#### TGA

**Fig. 8:**
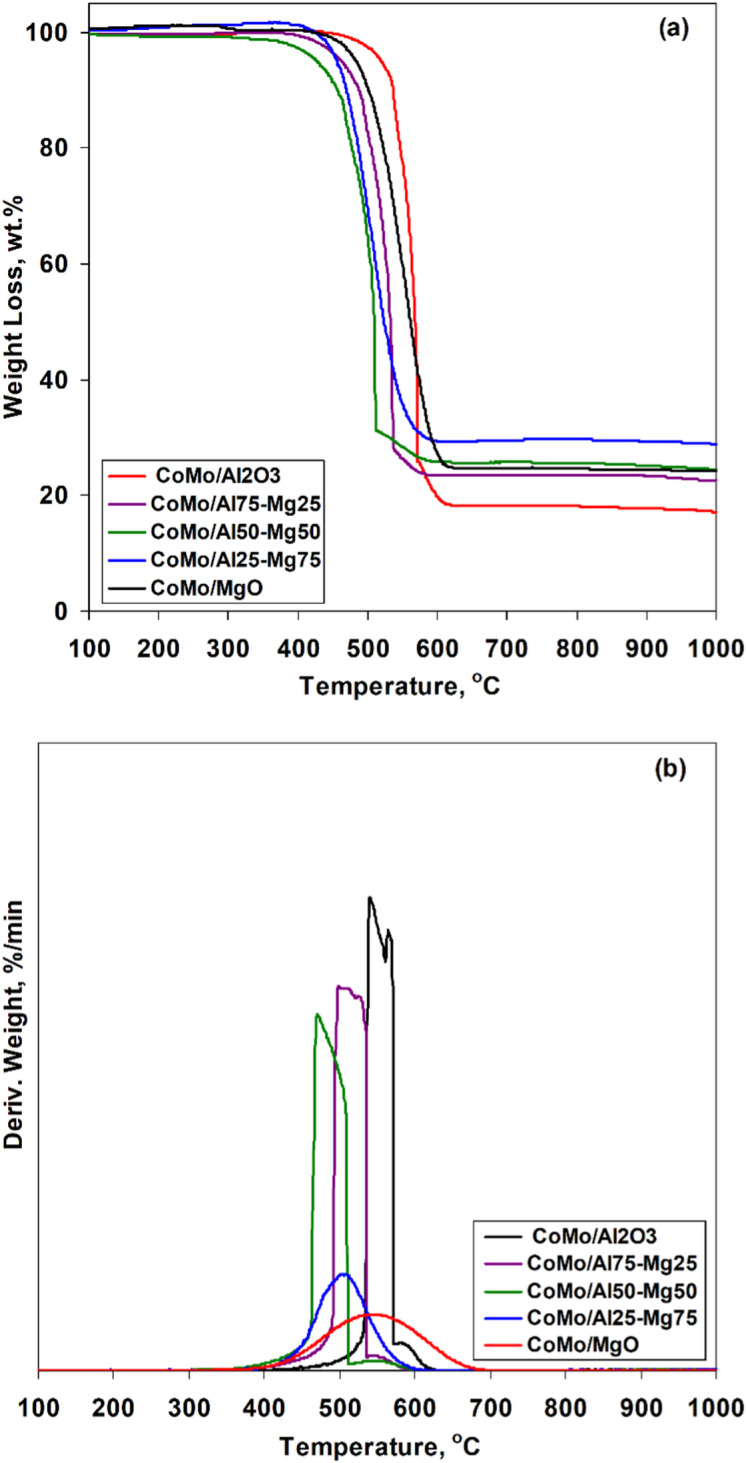
**(a)** TGA profiles **(b)** DTG Profiles for spent catalysts.

Through TGA and DTG informative analysis, the thermal stability of the as-deposited CNMs over the catalysts CoMo/Al_2_O_3_, CoMo/Al_75_-Mg_25_, CoMo/Al_50_-Mg_50_, CoMo/Al_25_-Mg_75_, and CoMo/MgO is investigated. Figure 8a displays the TGA plots for the used catalysts. It is evident that all as-grown CNMs degrade in a single step, with the majority of weight losses occurring between 450 and 600 °C. These degradation mechanisms ensure the crystalline nature of the as-deposited CNMs. Amorphous carbon is completely absent from all developed CNMs, as shown by the fact that there are no weight losses for any of the CNMs before 400 °C for all as-produced CNMs^11–13^. For all the produced CNMs, the onset, oxidation, offset, and onset-offset differences are listed in Table 2. Except for CoMo/Al_50_-Mg_50_, all the produced CNMs exhibit high thermal stability. CoMo/Al_50_-Mg_50_ spent catalyst exhibits the lowest oxidation temperature with a significant offset-onset difference. It was reported that the higher oxidation temperature with a low offset-onset difference signifies more graphitized CNMs^38^.

**Table 2:** TGA data of the as-grown CNMS over CoMo catalysts supported over bure and binary supports.

Composition	Onset temp., °C	Oxidation temp., °C	Offset temp., °C	Difference
CoMo/Al_2_O_3_	529	559	573	44
CoMo/Al_75_ – Mg_25_	480	511	537	57
CoMo/Al_50_ – Mg_50_	451	469	575	124
CoMo/Al_25_ – Mg_75_	454	503	553	80
CoMo/MgO	486	552	585	99

**Fig. 9:**
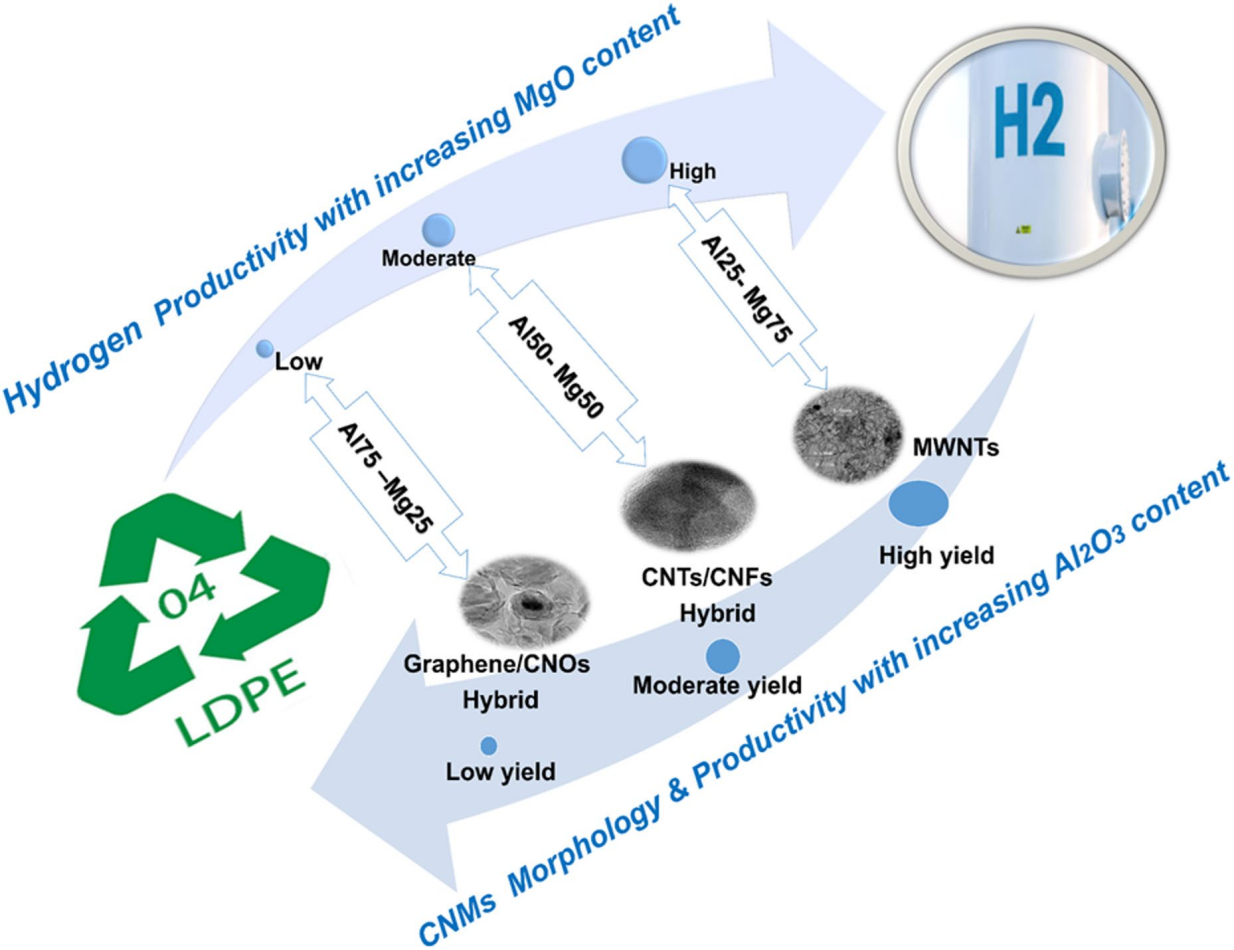
Influence of Al-Mg composition on H_2_ yield and CNMs structure and morphology.

DTG carves are depicted in Fig. 8b, confirming the variety of their oxidation peaks’ intensity, breadth, position, and number. As a result, not only is thermal stability deviation presented, but also purity degree is distinguished. Depending on the purity term, some samples might contain comparable crystalline CNMs with varying levels of purity. This information agrees with TEM findings, Raman signals, and XRD predictions. It might be assumed that CoMo/Al_2_O_3_ and CoMo/MgO catalysts produce more thermally stable, crystalline, and pure CNMs than CoMo/Al_2_O_3_-MgO catalysts as their inflection temperatures are lowered^17^. Moreover, it can be summarized that variations in the oxidation behaviors of the deposited CNMs are related to their structure and morphology, which are influenced by the nature and composition of their supports. Based on fresh and spent catalysts data, various CNMs growth path ways are designated in imaginative proposal to be important growth guidelines (Supplementary Figs. S7). To compare the present results, Table 3 demonstrates recent studies in this field. Figure 9 demonstrates the influence of different Al-Mg composition upon the hydrogen yield and the developed CNMs structure and morphologies.

**Table 3:** Recent studies for coproducing H_2_ and CNMs.

Catalyst	Feedstock	Technique	Hydrogen yield	Carbon nanomaterial yield	Carbon nanomaterial morphology	Ref.
Fe/γ-Al _2_O_3_	real-world waste plastics	Pyrolysis	22.9 mmol H _2_/g plastic)	195 mg g^− 1^ plastic	CNTs	^67^
Ni-Fe/γ-Al_2_O_3_	real-world waste plastics	Pyrolysis with steam	31.8 mmol H_2_/g plastic	287 mg g^− 1^ plastic	CNTs	^67^
Ni-Fe bimetallic catalyst	waste plastic	Pyrolysis	3.93 vol%	84.72 mg g^− 1^ plastic	CNTs	^68^
Ni-Mn-Al	waste polypropylene	Pyrolysis -reforming	71.4 mmol hydrogen g^− 1^ plastic	23 wt%	CNTs	^69^
10% Ni/Al2O3 10% Fe/Al2O3 10% Co/Al2O3	real-world polyethylene plastic film	Microwave	86.3 vol% 92 vol% 90.5 vol%.	70.5% 74.8% 73.1%	CNTs/CNFs	^70^
FeMo@Al2O3	waste tea residue and waste plastics	Catalytic co-pyrolysis.	49.57% (H_2_-rich syngas)	84.42% to catalyst	CNTs (mainly MWNTs)	^71^
Fe-Ni catalyst	Cellulose and polypropylene waste	Catalytic co-pyrolysis.	67 vol% (with CO)	40 wt%	CNTs	^72^
CoMo/MgO	LDPE	Two staged Catalytic decomposition	81 vol%	32.42 wt%	CNTs/CNFs	This work
CoMo/Al_25_ – Mg_75_	LDPE	Two staged Catalytic decomposition	72 vol%	26.14 wt%	MWNTs	This work
CoMo/Al_50_ – Mg_50_	LDPE	Two staged Catalytic decomposition	61.5 vol%	19.3 wt%	CNT/CNFs Bamboo CNTs (b-CNTs) and fishbone CNFs (f-CNFs)	This work
CoMo/Al_75_ – Mg_25_	LDPE	Two staged Catalytic decomposition	54.9 vol%	15.7 wt%	graphitic/carbon nano-onions (CNOs)	This work
CoMo/Al_2_O_3_	LDPE	Two staged Catalytic decomposition	69.8 vol%	18 wt%	graphitic / CNFs platelet-like CNFs (P-CNFs)/turbostratic CNFs (t-CNFs)	This work

## Conclusion

To elucidate the relationship between catalyst support composition, hydrogen production, and the structure, crystallinity, quality, and yield of carbon nanomaterials (CNMs), low-density polyethylene (LDPE) waste was employed as the feedstock in a continuous-flow catalytic pyrolysis system. CoMo catalysts supported on Al₂O₃, MgO, and Al₂O₃–MgO mixed-oxide supports with different compositions were systematically investigated. The textural and structural characteristics of the investigated supports indicated limited interactions between Al₂O₃ and MgO within the mixed-oxide matrices. The physicochemical properties of the fresh CoMo catalysts were governed by the individual characteristics of Al₂O₃ and MgO as well as by the composition of their mixed-oxide supports. Characterization of the fresh catalysts by FTIR, XRD, Raman spectroscopy, and H₂-TPR suggested a stronger interaction of MgO with the Co and Mo active species than that of Al₂O₃, resulting in a greater proportion of readily reducible and accessible active species. Although the formation of MgAl₂O₄ was not conclusively confirmed, its possible presence in the Al₂O₃–MgO-supported catalysts may have contributed to the observed reduction behavior. Consistent with these observations, CoMo/MgO exhibited the highest hydrogen-production performance, followed by CoMo/Al_25_–Mg_75_ and CoMo/Al₂O₃, whereas CoMo/Al_50_–Mg_50_ and CoMo/Al_75_–Mg_25_ showed lower catalytic activity. The lower catalytic performance of the latter catalysts is likely associated with the formation of less active segregated Co₃O₄, bulk CoMoOₓ, and inactive CoAl₂O₄ phases arising from the combined structural effects of Al₂O₃ and MgO. Furthermore, the pronounced agglomeration observed for CoMo/Al_75_–Mg_25_ and CoMo/Al_50_–Mg_50_ suggests weaker metal–support interactions and partial suppression of the molybdenum-assisted dispersion of cobalt species, thereby reducing the number of accessible active sites for hydrocarbon decomposition and hydrogen production. Among the CoMo/Al₂O₃–MgO mixed-oxide catalysts, CoMo/Al_25_–Mg_75_ exhibited the highest hydrogen concentration, indicating that increasing the MgO content in the mixed support enhances the availability of catalytically active sites and promotes hydrogen production. Although CoMo/MgO exhibited the highest hydrogen concentration overall, the superior performance of CoMo/Al_25_–Mg_75_ among the mixed-oxide catalysts highlights the beneficial role of MgO enrichment in optimizing catalytic activity while maintaining the synergistic effects of the mixed support. The deposited carbon nanomaterial (CNM) morphologies indicate that the CoMo/MgO and CoMo/Al_25_–Mg_75_ catalysts preferentially promoted the formation of tubular carbon nanostructures with well-graphitized walls. In contrast, the CoMo/Al₂O₃, CoMo/Al_75_–Mg_25_, and CoMo/Al_50_–Mg_50_ catalysts favored the formation of randomly organized turbostratic carbon together with a variety of CNM morphologies, including carbon nano-onions (CNOs), bamboo carbon nanotubes (b-CNTs), and densely stacked graphitic flakes. Collectively, these morphologies suggest a lower degree of graphitization and structural order than those obtained over CoMo/MgO and CoMo/Al_25_–Mg_75_. Moreover, the formation of these disordered carbon structures is likely associated with the catalyst deactivation observed during the reaction. In contrast, the predominance of homogeneous multi-walled carbon nanotubes (MWCNTs) over CoMo/Al_25_–Mg_75_ indicates the formation of carbon nanomaterials with a higher degree of graphitization and structural uniformity. This morphology is consistent with the sustained catalytic activity and prolonged hydrogen production observed under the investigated conditions, although the superior hydrogen-production performance of CoMo/MgO indicates that carbon morphology represents one of several factors governing the overall catalytic behavior.

## Supplementary Information

Below is the link to the electronic supplementary material.

Supplementary Material 1

## Data Availability

All data that support the findings in this study are available in the article. Additional information is available from the corresponding author upon request.
