# Supplementary material for: Dual production of turquoise hydrogen and hybrid carbon nano materials from plastic waste over Co-Mo/Al-Mg catalysts
Source: Sci Rep. 2026 Aug 19;16:26027. doi: 10.1038/s41598-026-66503-3 (PMC13487191; doi:10.1038/s41598-026-66503-3)
Supplement: Supplementary file 1 [file 41598_2026_66503_MOESM1_ESM.doc]

**Supplementary data**

**Experimental**

***Support preparation***

Three supports, known as Al_75_-Mg_25_, Al_50_-Mg_50_, and Al_25_-Mg_75_, were produced using composites of Al_2_O_3_ and MgO with weight ratios of 3:1, 1:1, and 1:3. The calculated weights of Al(NO_3_)_3_.H_2_O and Mg(NO_3_)_2_.H_2_O are typically dissolved in the proper volumes of deionized water, and their aqueous solutions are then combined and stirred at 90°C to evaporate their water. A full day of drying at 120°C was done on the final pastes. Following that, the dry materials were crushed and heated for four hours at 500°C.

***Procedure setup for hydrogen and CNMs co-generation process***

A single run would be utilized to co-produce hydrogen and CNMS from LDPE waste using a two-stage atmospheric continuous flow system [42]. Our earlier efforts Aboul-Enein and Awadallah [39] reported the specific compartments of the employed system. The smallest LDPE waste fragments, weighing 15 g, were typically placed in the first reactor, while each manufactured CoMo-based catalyst, weighing about 0.5 g, was introduced in the second reactor. In fact, the temperatures of the first LDPE thermal cracking furnace and the second gaseous hydrocarbon catalytic cracking furnace were adjusted at 500 and 700°C, respectively. Each run starts with a reduction of the catalyst at 700°C for an hour. After turning on each system compartment, the trial was then run for 150 minutes. During the reaction period, the produced gas was routed to a PerkinElmer Clarus 500 online gas chromatography instrument that was outfitted with flame ionization and thermal conductivity detectors using Rt-Alumina BOND/Na_2_SO_4_ capillary column (30m-0.53mm ID (GC) for detecting and evaluating the hydrogen gas concentration in relation to other gaseous hydrocarbons. After completing the reaction period, furnaces were turned off, valves were adjusted again, and the finished CNMs were cooled using a nitrogen flow. Using the following formula, the carbon yield might be calculated in relation to the quantity of fresh catalyst.

Carbon yield (%)=(W_S.Cata_.–W_F.Cata_.)/W_F.Cata_. X100, Eq. (1)

Where, W_S_. _Cata._ is the total weight of spent catalyst (catalyst and as-deposited CNMs) and W_F. Cata._ is the weight of the fresh catalyst.

**S.1 Result and discussion**

***S.1.1 Characterization of the prepared supports***

*S.1.1.1 FTIR*

Fig. S1 depicts the FTIR spectra for Al_2_O_3_, MgO, Al_75_-Mg_25_, Al_50_-Mg_50_, and Al_25_-Mg_75_ binaries to show how much magnesia and alumina interact in their binary oxides. Adsorbed molecules such as water, carbonates, and carbon dioxides are typically found [30]. According to supplementary Fig. S1, bands between 500 and 1000 cm^-1^ are caused by the stretching vibration of Al-O-Al bonds. Octahedral coordinated units of AlO6 are observed in Al_2_O_3_ by bands at 600, 658, and 741 cm^-1^ [S1]. MgO has its distinctive band at about 429 cm^-1^ [S2]. On introducing both Al_2_O_3_ and MgO in their binary composites, their bands are diminished. Additionally, for Al75-Mg25, a very faint Al-O-Mg band is evolved at about 850 cm^-1^ [26]. This demonstrates that Al_2_O_3_ and MgO have little interaction. Al50-Mg50 also shows two faint bands at around 530 and 695 cm^-1^, which may indicate that MgAl_2_O_4_ formed at least in part [35]. Al_25_-Mg_75_ also detects a band at approximately 881 cm^-1^ that can be attributed to tetrahedral coordinated alumina units (AlO_4_) [S1]. The evidence provided above clearly shows that weak amounts of interacting Al-Mg(O) species exist in all mixed oxides due to inefficient calcination temperature.

*S.1.1.2 XRD*

Supplementary Fig. S.2 illustrates XRD patterns of the calcined MgO, Al_2_O_3_, and Mg-Al mixed oxide supports. Because of the extremely weak diffraction phase of ɤ-Al_2_O_3_ at 31.5° and the impurity boehmite (AlOOH), the bare Al_2_O_3_ support has a fairly crystalline structure [28]. Since there are no discernible crystalline Al_2_O_3_ peaks, all Mg-Al mixed oxides have amorphous Al_2_O_3_ structures. On the other hand, for pure MgO support, five distinctive cubic MgO peaks (2Ө = 43°, 37°, 62.4°, 74.8°, and 78.6°) have been identified. Rationally, as the Al_2_O_3_ content in the Mg-Al mixed oxides increases, the MgO peaks' intensities decrease and eventually vanish in Al75-Mg25. As a result, it is possible to infer that adding Al_2_O_3_ to MgO reduces the crystallinity of the resulting Mg-Al mixed oxide. Additionally, adding MgO to Al_2_O_3_ prevents it from transforming into a crystalline phase [27]. Al25-Mg75 and Al50-Mg50 mixed oxides exhibit a slight shift of MgO phases to lower diffractions (43° to 42.7° and 62.4° to 62°, Fig. S.2). There is no chance of the formation of spinel MgAl_2_O_4_ or (Mg, Al)O solid solutions, which were reported to form at higher calcination temperatures [23]. Furthermore, it was claimed that Mg(Al)O solid solution Mg-Al mixed oxide diffractions were likely to resemble MgO diffractions [29].

*S.1.1.3 BET*

Supplementary Table S1 lists measurements of specific surface area, pore volume distribution, and average pore diameter that describe the textural characteristics of fresh Al_2_O_3_, MgO, and their binary oxides. It is clear that Al_2_O_3_ has the highest surface area (148.6 m^2^/g), while pure MgO has the lowest surface area (9 m^2^/g). Incorporating MgO into Al_2_O_3_ has a detrimental effect, resulting in 16.9 m^2^/g and 0.039 cc/g in Al_75_-Mg_25_ binary oxide. Highly dispersed MgO in Al_2_O_3_ would plug their pores and reduce their surface area. According to Krishnan et al. [S3], MgO's hygroscopic properties caused it to have a smaller surface area. On the other hand, adding Al_2_O_3_ to MgO improves the surface characteristics of their binary oxides, Al_25_-Mg_75_ and Al_50_-Mg_50_, to 86.3 m^2^/g and 97 m^2^/g, respectively. By preventing MgO segregation, Al_2_O_3_ functions as a structural promoter.

Further explorations of textural properties are carried out for the prepared calcined CoMo-based catalysts through nitrogen adsorption and desorption measurements. Supplementary Table S2 highlights clear conversions between supports and catalysts in specific surface areas (S_BET_), average pore diameter, and total pore volume. Excluding the CoMo/MgO and the CoMo/Al_75_-Mg_25_ catalysts, all of the prepared catalysts exhibit lower S_BET_ values than their supports, which may be due to pore blocking [23,S4]or an accumulation of bulk Co_3_O_4_ rather than (Co, Mo, Al, Mg) interacting species [18]. Even though adding MgO to Al_2_O_3_ for the Al75-Mg25 support has a negative effect (Supplementary Table S1), the opposite of this trend is examined for the CoMo/Al75-Mg25 catalyst (Supplementary Table S2). Because of the higher affinity of the incorporated MgO for the impregnated bi-metals, particularly molybdenum, both Co_3_O_4_ and MoO_3_ may be transferred from the catalyst surface to the subsurface layers of the support, forming MgCo_2_O_4_ and MgMoO_4_, respectively. Another possibility is that the added bimetals could remove the dispersed MgO from the Al_2_O_3_'s interior layers and transport it to the surface, leaving pores and cavities [52]. Choya et al. [23] concur with this finding and propose that the MgO-Al_2_O_3_ interaction resulted in the formation of narrower pores, contributing to the total specific area. Particularly noteworthy is the sharp increase in S_BET_ for CoMo/MgO catalysts compared to bare MgO supports, which is associated with (Co, Mo, MgO) interacting species [12]. According to a comparison of the different catalysts, CoMo/Al_2_O_3_ and CoMo/Al75-Mg25 catalysts have the highest specific surface areas, while CoMo/MgO catalyst has the lowest. Because MgO has a small surface area, obtaining a larger specific surface requires using lower magnesia content [16]. Additionally, it was reported that adding molybdenum catalyzed the creation of MgAl_2_O_4_ spinel and increased the calcination temperature to 600°C [52], which was in agreement with our XRD results. Özdemir and Öksüzömer [35] stated that loading metals on MgAl_2_O_4_ diminishes the specific surface area by forming larger pore volumes. It is in line with the hypothesis that rising MgO content would result in an increase in total pore volume (Table S2).

***S.1.2 Effect of alumina-magnesia mixed oxides supported Co-Mo catalysts upon product composition***

Supplementary Table S3 summarizes the catalytic reactivity of each catalyst with regard to mass balance calculations. When taken into consideration, these calculations depend upon the conversion values of gaseous hydrocarbon inlets that are emitted from the first stage (the LDPE thermal cracking and cooling unit) to the second stage of catalytic cracking during 150 minutes. Only the CoMo/MgO catalyst has prolonged reactivity to operate the best catalytic behavior throughout the reaction progression when compared to the complete catalytic decomposition of inlet gaseous hydrocarbons to carbon, hydrogen, and methane. A further portion of higher hydrocarbon gases that reach 7.2 Vol.% results from loading Al_2_O_3_ into MgO (over CoMo/Al25-Mg75) because the catalyst is unable to completely decompose the inlet gases to carbon, hydrogen, and methane. However, due to the higher yield of C_+2_ hydrocarbons (6.9 vol.%), CoMo/Al_2_O_3_ exhibits less catalytic activity of the inlet hydrocarbons than CoMo/MgO and CoMo/Al_25_-Mg_75_. Similarly, adding MgO to CoMo/Al_2_O_3_ catalysts causes them to lose catalytic efficiency, resulting in the lowest conversion of the inlet gaseous hydrocarbons. For CoMo/Al_75_-Mg_25_ and CoMo/Al_50_-Mg_50_, the volumetric content of C_+2_ hydrocarbons have increased to 21.3 and 14.5 Vol. %, respectively. Thus, the catalytic efficiency of CoMo with bare MgO and bare Al_2_O_3_ is higher than that of their binary oxides, such as Al_75_-Mg_25_ and Al_50_-Mg_50_.

Based on hydrogen output; CoMo/MgO exhibits a higher hydrogen concentration than CoMo/Al_2_O_3_. Increasing the magnesia content in their binary mixed oxides raises the hydrogen concentration via CoMo/Al_75_-Mg_25_ from 54.9 to 61.5 by CoMo/Al_50_-Mg50 and to 72 Vol.% with CoMo/Al_25_-Mg_75_. Because the catalyst can further catalyse the resultant methane's breakdown into carbon and hydrogen, CoMo/MgO exhibits the lowest concentration of the resulting methane [S5]. According to CNMs yield, CoMo/MgO and CoMo/Al_25_-Mg_75_ catalysts have the highest CNMs deposition efficiency while CoMo/Al_2_O_3_ and CoMo/Al_75_-Mg_25_ catalysts have the lowest CNMs yield. The CoMo/Al_50_-Mg_50_ catalyst has a moderate CNMs yield that is in the middle of the CoMo/Al25-Mg75 and CoMo/Al_75_-Mg_25_ catalysts.

***S.1.3 Characterization of the as-grown CNMs structures***

*S.1.3.1 HR-TEM*

TEM images are carefully captured at various scales of magnification to investigate the macro and microstructures as well as the nature and types of the as-deposited carbon nanomaterials (CNMs) on catalysts with various support combinations of alumina and magnesia. Supplementary Figs. S3-5 depict variations in the typical morphologies of each used catalyst.  It's interesting to note that, on single catalyst samples, an intriguing hybrid structure of CNMs is created by the combination of various CNMs structures and morphologies. Meanwhile, variation in the composition of support has a great influence on the nature and type of the developed active sites and their activities. Furthermore, it is readily apparent that there is a difference and deviation in the levels of crystallinity and graphitization in the CNMs produced for various catalyst compositions. Therefore, these photos could indicate the quality of the as-grown CNM_S_.

In the case of bare supported catalysts, CoMo/Al_2_O_3_ develops a composite of graphitic and CNFs (Supplementary Figs. S. 3A–D), whereas in CoMo/MgO, a hybrid of CNTs/CNFs is scrutinized (Supplementary Figs. S. 3E–F). In fact, different-shaped CNFs have been found in both catalysts. CoMo/MgO exhibits ribbon CNFs (r-CNFs) that resemble ribbon CNTs (r-CNTs) and have parallel graphitic layers to the axis (Supplementary Fig. S. 3E-F). On the other hand, for CoMo/Al_2_O_3_, Fig. S. 3A–D shows platelet-like CNFs (P-CNFs)/turbostratic CNFs (t-CNFs). According to Supplementary Fig. S. 3A-B, graphitic layers are most likely stacked perpendicularly to the fiber's axis center, resembling the P-CNFs configuration [S6]. Despite the ordered graphitic layer mode, the graphitic layers are stacked irregularly to create turbostratic materials (t-CNFs). Therefore, it is clear that there is a difference in the quality, graphitization, and heterogeneity of the produced CNMs produced on both bare MgO and Al_2_O_3_ supported CoMo catalysts. Another composite of graphitic/carbon nano-onions (CNOs) is produced by CoMo/Al75-Mg25 (Supplementary Figs. S. 4A-D). Extra-large metallic agglomerate particles have been noticed, affirming the extremely weak metal-support interactions. Therefore, the addition of MgO lessens the interaction between the active species (Co, Mo) and their support (Al_2_O_3_), speeds up the sintering of cobalt particles, and declines the distributions of active sites. Additionally, the deposition of graphitic layers could be proof of releasing cobalt mat [66], acting as a graphitic layers template. Low-transparency graphitic layers, as seen in Fig. 10C-D, signify the deposition of more graphitic layers that overlapped one another. Further efforts [39] supported the idea that higher transparent graphitic sheets indicated a lower number of deposited graphene sheets and confirmed their separated manner from metallic particles. It is remarkable to spot encapsulated cobalt species with graphitic layers in the core of CNOs [19], as well as the dissipation of cobalt particles with dense graphitic layers as full covers (Supplementary Fig. S. 4C) [S7].

Contrarily, according to Supplementary Fig. S.4E-F, adding Al_2_O_3_ to MgO-supported catalyst, CoMo/Al_25_-Mg_75_, increases the homogeneity of the deposited CNMs to produce MWNTs [64] with a narrower diameter than those made by CoMo/MgO (Supplementary Fig. S.3E). Therefore, a small amount of added Al_2_O_3_ enhances the dispersion of active sites by preventing the agglomeration of non-interacting Co_3_O_4_. Rationally, the CoMo/Al_50_-Mg_50_ catalyst would have synergetic structural influences from both binders, Al_2_O_3_ and MgO. For CoMo/Al_50_-Mg_50,_ Supplementary Fig. S.5 depicts a composite of CNT/CNFs in various configurations. Bamboo CNTs (b-CNTs) would be affected by active sites that are dispersed in MgO, whereas turbostratic CNFs with a conflicting graphitic orientation and a rugged surface would be related to Al_2_O_3_ influence. Clearly shown in Supplementary Fig. 3.5F, the alternative morphology of developed CNFs type's microstructure is one in which the graphitic walls slope diagonally to the middle fiber axis with a distinct angle and cone. These CNFs are known as fishbone CNFs (f-CNFs) or herringbone CNFs because they resemble the skeleton of a fish [S.6]. Additionally, f-CNFs would be turbostratic materials if the cones of graphitic sheets are deposited randomly without a clear arrangement (Supplementary Fig. S.5C–D). It is made clear that there are distorted CNMs and large metallic particles present (Supplementary Fig. S.5A-B). On the other hand, it is perceived that increasing MgO content to Al_2_O_3_ portion in their binary mixed oxides catalysts improves graphitization degree and homogeneity. Therefore, compared to catalysts with successive alumina contents, CoMo/MgO and CoMo/Al_25_-Mg_75_ deposit more regular and smoother CNMs with definite ordered of graphitic layers and clear homogenous morphology.

*S.1.3.2 XRD*

Fig. S.6 depicts the XRD patterns of the used catalysts. The deposition of CNMs with graphitic structure is amply demonstrated by the presence of a dominant intense graphitization peak at approximately 26° [64] for all of the used catalysts. In addition, the disappearance of metal oxide phases confirms their conversion to the metallic phase at about 44° and 52° and carbide forms at around 39°. For comparison, it is obvious to note decreasing intensity of graphitization peak with successive loading MgO. This may be related to the nature of the CNMs produced. However, many researchers reported that the more crystalline a structure is formed efforts [39], the more CNMs are grown [36], and the more intense and sharper a graphitic peak develops. The peak intensity may also depend on the type of CNMs being grown. As a result, CNTs would not increase the peak intensity as much as larger diameter CNFs and concentrated graphitic flakes would. Awadallah et al. [10] also stated that larger diameter CNTs with dense graphitic walls would result in a sharper graphitic peak. Therefore, it could be concluded that in the cases of CoMo/Al_2_O_3_, CoMo/Al_75_-Mg_25_, and CoMo/Al_50_-Mg_50_ catalysts, high deposition of graphitic layers on large aggregated nanoparticle crystallites may explain the development of intensive graphitic peaks. On the other hand, the less intense graphitic peaks for the CoMo/MgO and CoMo/Al_25_-Mg_75_ catalysts may be the result of MWNTs having fewer graphitic walls. From the perspective described above, XRD data would agree with both the Raman and the TEM outlines.

S.1.4 Effect of CNMs structure on the catalyst activity toward hydrogen production

To evaluate the activity of the investigated catalyst, it is important to correlate the nature and morphology of the as-deposited CNMs of each catalyst with its hydrogen output. Many works of literature attribute the catalyst deactivation for hydrogen production to the accumulation of CNMs or amorphous carbon's influence on pore blocking, while others believe that hydrogen generation is proportional to the growth of CNMs. Accordingly, the dominant key is the geometry of the as-deposited nanomaterial, which may have many catalytic effects. Indeed, both hydrogen and elemental carbon are emitted from the adsorption and catalytic decomposition of hydrocarbons. Thereby, continuous adsorption and decomposition availability on the catalyst surface would lead to a higher concentration of hydrogen and elemental carbon. The surface properties of the catalyst and surface processes would be affected by the reconstruction of carbon atoms through additional processes into the catalyst bulk, such as diffusion, precipitation, and nucleation.

According to the characterization of the used catalyst, the growth of tubular morphologies like CNTs and CNFs does not impair the catalytic performance for adsorbing and converting additional hydrocarbons to hydrogen and carbon in the case of CoMo/MgO and CoMo/Al_25_-Mg_75_. In CNMs with a tubular structure, cobalt nanoparticles are maintained active and ready, prolonging the catalytic activity of the particles throughout the entire reaction process. In contrast, the accumulation of flat, dense graphitic layers on extra-large cobalt nanoparticles indicates the acceleration rate of the bulk process, which covers the cobalt nanoparticles and wouldn’t be in contact with further inlet hydrocarbons. Likewise, the conformation of CNOs may trap cobalt nanoparticles with circular graphene layers, halting their surface reaction. These CNMs' morphologies could cause the lowest catalytic activity for CoMo/Al_75_-Mg_25_. In addition, CNMs with turbostratic configurations, which are produced on CoMo/Al_2_O_3_ and CoMo/Al_50_-Mg_50_, would have a poisonous influence on the catalytic activity.

***S.1.5 Assumptions of CNMs growth routes***

Based on fresh and spent catalysts data, various CNMs growth path ways are designated in imaginative proposal to be important growth guidelines (Supplementary Fig. S.7). For bare supported catalysts, r-CNTs/CNFs hybrid formation ensures stronger interaction between MgO and Co_3_O_4_ and MoO_3_ rather than Al_2_O_3_ which are proved by XRD and Raman. So, aggregation of Co_3_O_4_ particles to larger particle size is responsible for growth of p-CNFs, t-CNFs and graphitic flakes. Supplementary Fig. S.7a illustrates poor dispersion of Co_3_O_4_ particles on bare alumina surface and simplifies each structure features. Moreover, this rapid precipitation rate of graphitic platelets may cause disordering in growth directions yielding t- CNFs (Supplementary Fig. S.7a). As above-mentioned, the morphology of the produced CNMs is reversible to the shape of metallic particles. It was reported that peer like particle with cone results in tubular CNFs where, cubic like particle grows p-CNFs and t-CNFs deposits on rough-shapeless particle. Developing CoMoO_4_, MgCo_2_O_4_and MgMoO_4_ not only distributes Co_3_O_4_ but also hinders sintering of their molecules. So, they have a dominant role to grow regular CNF_S_ and CNTs (Supplementary Fig. S.7e).

Additionally, formation of graphitic/CNOs hybrid reveals the structural barrier role of the incorporated MgO which is in agreement with Raman, TPR and XRD. This barrier enhances sintering of Co_3_O_4_ to extra-large particle size and promotes the interaction between Co_3_O_4_ and MoO_3_ (Supplementary Fig. S.7b). The presence of large agglomerated particles of CoMoO_x_ species may be elongated in quasi-liquid state and may grow graphitic platelets. Also, extra-large aggregated Co_3_O_4_ particles during calcination, reduction and reaction stream, maybe decorated to metallic sheet as a result of extremely weak metal-support interaction which function as graphitic template (Supplementary Fig. S7.b). In addition, incorporating MgO may enhance the aggregation of CoMoO_x_ building unites which could be expanded in different orientations in quasi-liquid state and could be reshaped in mat shape-like. These mats may stack successive graphitic platelets (Supplementary Fig. S.7b). In contrast to CoMo/Al_75_-Mg_25_ catalyst, loading Al_2_O_3_ to MgO as a binder, CoMo/Al_25_-Mg_75_ catalyst, homogenous narrower CNTs defines highly dispersed smaller cobalt nanoparticles (Supplementary Fig. S.7d) demonstrates the structural role of Al_2_O_3_ which functions as dispersing agent for Co_3_O_4_ particles. Increasing the homogeneity of metallic cobalt particle size leads to highly uniform active sites distribution, limits aggregation of metal particles and produces better CNT graphitization with narrow diameter distribution. Supplementary Fig. S.7c demonstrates diagrammatically the influence of equivalent content of Al_2_O_3_ and MgO on the active sites’ nature. Theoretically, it is assumed two main regions for CoMo/Al50-Mg50 with their developed species suchlike different particle size of Co_3_O_4_ and mixed oxides of Co, Mo, Al_2_O_3_ and MgO. Interestingly, synergistic characters of both MgO and Al_2_O_3_ would be emerged to release composite of b-CNTs/f-CNFs with little t-CNFs. Compared to f-CNFs, growth of ribbon CNFs results from lower decomposition rate than surface diffusion.

**References**

[S1] A. Adamczyk, E. Długon´,The FTIR studies of gels and thin films of Al_2_O_3_–TiO_2_ and Al_2_O_3_–TiO_2_–SiO_2_ Systems, Spectrochimica Acta Part A 89 (2012) 11– 17.

[S2] P. Gao, X. Tian, C. Yang, Z. Zhou, Y. Li, Y. Wang, S. Komarneni, Fabrication, performance and mechanism of MgO meso-/macroporous nanostructures for simultaneous removal of As (iii) and F in a groundwater system, Environ. Sci. Nano. 3 (2016) 1416–1424.

[S3] S. G. Krishnan, M.V. Reddy, M. Harilal, B. Vidyadharan, I. I. Misnon, M. H. A. Rahim, J. Ismail, R. Jose, Characterization of MgCo_2_O_4_ as an electrode for high performance supercapacitors, Electrochimica Acta 161 (2015) 312–321.

[S4] N. D. Charisiou, K. N. Papageridis, L. Tzounis, V. Sebastian, S. J. Hinder, M. A. Baker, M. AlKetbi, K. Polychronopoulou, M. A. Goula, Ni supported on CaO-MgO-Al_2_O_3_ as a highly selective and stable catalyst for H_2_ production via the glycerol steam reforming reaction, international journal of hydrogen energy 44 (2019) 256-273.

[S5] G. S. Bajad, R.P. Vijayakumar, A. G. Gupta, V. Jagtap, Y. pal Singh, Production of liquid hydrocarbons, carbon nanotubes and hydrogen rich gases from waste plastic in a multi-core reactor, Journal of Analytical and Applied Pyrolysis 125 (2017) 83–90.

[S6] I. U. Dina, M. S. Shaharunb, A. Naeemc, M. A. Alotaibia, A. I. Alharthia, M. A. Bakhta, Q. Nasir, Carbon nanofibers as potential materials for catalysts support, a mini-review on recent advances and future perspective, Ceramics International 46 (2020) 18446–18452.

[S7] P. Jana, V. A. de la Pen͂a O’Shea, J. M. Coronado, D. P. Serrano, Co-production of graphene sheets and hydrogen by decomposition of methane using cobalt based catalysts†, Energy & Environmental Science 4 (2011) 778 –783.

**Tables**

|  | **Supplementary Table S1:** Surface properties for bare and their binary oxides supports. | | | |
| --- | --- | --- | --- | --- |
| DP (nm) | | VP (cc/g) | S_BET_ (m^2^/g) | Support |
| 3.69 | | 0.225 | 148.6 | Al_2_O_3_ |
| 2.35 | | 0.039 | 16.9 | Al75 – Mg25 |
| 3.5 | | 0.214 | 97.2 | Al50 – Mg50 |
| 6.2 | | 0.151 | 86.3 | Al25 – Mg75 |
| 21.7 | | 8.8 | 7.9 | MgO |

|  | **Supplementary Table S2:** Surface properties and average crystallite size for fresh calcined Co-Mo catalysts supported over bare and binary supports. | | | | |
| --- | --- | --- | --- | --- | --- |
| Average crystallite size | | DP (nm) | VP (cc/g) | S_BET_ (m^2^/g) | Catalyst |
| 19.67 | | 3.858 | 0.150 | 70.07 | CoMo/Al_2_O_3_ |
| 20.79 | | 1.603 | 0.117 | 77.23 | CoMo/Al75 – Mg25 |
| 22.6 | | 3.671 | 0.164 | 63.18 | CoMo/Al50 – Mg50 |
| 16.92 | | 3.708 | 0.163 | 56.76 | CoMo/Al25 - Mg75 |
| 20.56 | | 23.01 | 0.343 | 53.86 | CoMo/MgO |

| **Supplementary Table S.3:** Typical product compositions of two stage catalytic decomposition of LDPE waste over Co-Mo catalysts supported over bare and binary oxides supports during TOS 150 minute. | | | | | | |
| --- | --- | --- | --- | --- | --- | --- |
| C_2+_  Vol.% | CH_4_  Vol. % | H_2_ Vol.% | Gases  wt.% | | CNM  Wt.% | Catalyst |
| 6.9 | 23.3 | 69.8(±10) | 82 | 18 | | CoMo/Al_2_O_3_ |
| 21.3 | 23.8 | 54.9(±3.9) | 84.3 | 15.7 | | CoMo/Al75–Mg25 |
| 14.5 | 24 | 61.5(±3) | 80.7 | 19.3 | | CoMo/Al50–Mg50 |
| 7.21 | 21 | 72(±1.8) | 73.86 | 26.14 | | CoMo/Al25– Mg75 |
| 0 | 19 | 81(±1.4) | 67.58 | 32.42 | | CoMo/MgO |

**Figures**

**Supplementary Fig. S.1:** FTIR spectra of bare and binary supports.

**Supplementary Fig. S.2:** XRD patterns of calcined supports.

**supports.**

and binary supports.

**Supplementary Fig. S.3:** TEM photos for as-grown CNMs over CoMo catalysts: **(A-D)** for CoMo/Al_2_O_3_ and **(E-F)** for CoMo/MgO.

**Platelet Carbon nano-fiber**

**Turbostratic materials**

**Carbon**

**nano-fibers**

**Carbon**

**nano-tube**

**Supplementary Fig. S.4:** TEM photos for as-grown CNMs over CoMo catalysts: **(A-D)** for CoMo/Al_75_-Mg_25_ and **(E-F)** for CoMo/Al_25_-Mg_75_.

**Agglomerated**

**catalyst**

**Carbon nano-onion**

**Graphitic layers**

**Carbon nano-tubes**

**Supplementary Fig. S.5:** TEM photos for as-grown CNMs.

over CoMo/Al_50_-Mg_50_.

**Bamboo Carbon**

**nano-fiber**

**Turbostratic materials**

**Fishbone carbon nano-fiber**

**Supplementary Fig. S.6:** XRD data of as-grown CNMs.

**Supplementary Fig. S.7:** Novel assumption of as-grown CNMs routes. **Supplementary**
